# The Polar Lipidome of Cultured *Emiliania huxleyi*: A Source of Bioactive Lipids with Relevance for Biotechnological Applications

**DOI:** 10.3390/biom10101434

**Published:** 2020-10-12

**Authors:** Susana S. Aveiro, Tânia Melo, Ana Figueiredo, Pedro Domingues, Hugo Pereira, Inês B. Maia, Joana Silva, M. Rosário Domingues, Cláudia Nunes, Ana S. P. Moreira

**Affiliations:** 1 Mass Spectrometry Center, LAQV-REQUIMTE, Department of Chemistry, University of Aveiro, Campus Universitário de Santiago, 3810-193 Aveiro, Portugal; s.aveiro@ua.pt (S.S.A.); ana90@ua.pt (A.F.); p.domingues@ua.pt (P.D.); mrd@ua.pt (M.R.D.); 2 ECOMARE, CESAM—Centre for Environmental and Marine Studies, Department of Chemistry, University of Aveiro, Campus Universitário de Santiago, 3810-193 Aveiro, Portugal; taniamelo@ua.pt; 3 Green Colab—Associação Oceano Verde, University of Algarve, Campus de Gambelas, 8005-139 Faro, Portugal; galvaohugo@gmail.com; 4 Centre of Marine Sciences, University of Algarve, Campus de Gambelas, 8005-139 Faro, Portugal; ibmaia@ualg.pt; 5 Allmicroalgae Natural Products S.A., Apartado 9, 2449-909 Pataias, Portugal; joana.g.silva@allmicroalgae.com; 6 CICECO—Aveiro Institute of Materials, Department of Chemistry, University of Aveiro, Campus Universitário de Santiago, 3810-193 Aveiro, Portugal; claudianunes@ua.pt

**Keywords:** haptophyta, lipidomics, mass spectrometry, microalgae, *Emiliania huxleyi*

## Abstract

Polar lipids from microalgae have aroused greater interest as a natural source of omega-3 (*n*-3) polyunsaturated fatty acids (PUFA), an alternative to fish, but also as bioactive compounds with multiple applications. The present study aims to characterize the polar lipid profile of cultured microalga *Emiliania huxleyi* using hydrophilic interaction liquid chromatography coupled with high-resolution mass spectrometry (HILIC–MS) and fatty acids (FA) analysis by gas chromatography (GC–MS). The lipidome of *E. huxleyi* revealed the presence of distinct *n*-3 PUFA (40% of total FA), namely docosahexaenoic acid (22:6*n*-3) and stearidonic acid (18:4*n*-3), which give this microalga an increased commercial value as a source of *n*-3 PUFA present in the form of polar lipids. A total of 134 species of polar lipids were identified and some of these species, particularly glycolipids, have already been reported for their bioactive properties. Among betaine lipids, the diacylglyceryl carboxyhydroxymethylcholine (DGCC) class is the least reported in microalgae. For the first time, monomethylphosphatidylethanolamine (MMPE) has been found in the lipidome of *E. huxleyi*. Overall, this study highlights the potential of *E. huxleyi* as a sustainable source of high-value polar lipids that can be exploited for different applications, namely human and animal nutrition, cosmetics, and pharmaceuticals.

## 1. Introduction

Microalgae have attracted great interest in various biotechnological applications, including human and animal nutrition, cosmetics, and pharmaceuticals [1], representing a sustainable feedstock to supply new products and materials, as well as various high-value compounds. Furthermore, different strains of microalgae can grow rapidly in well-controlled production systems (e.g., photobioreactors) [2], allowing scaling to high volumes for large-scale industrial production of microalgal biomass.

Coccolithophores (classified under the phylum Haptophyta) are a large group of marine phytoplankton characterized by their external calcium carbonate plates, called coccoliths [3]. The coccolithophore, *Emiliania huxleyi,* is one of the most abundant and widely distributed microalgae in all oceans, except polar oceans. Although its key function in calcite production and the marine carbon cycle is well recognized [4], the commercial value of *E. huxleyi* has been poorly explored as a source of valuable compounds. To valorize the biomass of this microalga via biorefinery approaches, it is important to determine its biochemical composition and their bioactive and value-added components.

Lipids from microalgae are considered important nutrients, mainly marine strains are rich in omega-3 (*n*-3) polyunsaturated fatty acids (PUFA), especially docosahexaenoic acid (DHA, 22:6*n*-3) and eicosapentaenoic acid (EPA, 20:5*n*-3). These biomolecules have high scientific evidence on health benefits [5,6] and are generally obtained from fish oils. In particular, polar lipids from microalgae (not known for *E. huxleyi*) have emerged with potential interest, either as natural carriers of *n*-3 PUFA with high nutritional value or as bioactive compounds with multiple applications [7]. Despite their biological activities, such as antibacterial, antiviral, anti-inflammatory, and antitumor effects [8,9], the bioactivity of polar lipids depends on structural details such as the composition of fatty acyl and the polar head [2,3]. In this context, by addressing the valorization of the high-value polar lipids produced and thus improving the economic feasibility of the potential of cultured *E. huxleyi*, it is crucial to characterize the polar lipidome with the identification of individual lipid species.

Concerning the lipid composition of *E. huxleyi*, neutral long-chain lipids, including C_37–39_ alkenones (unsaturated ketones) and related alkenoates and alkenes, have received particular attention from the paleoclimatic community as indicators of past sea surface temperatures [10,11]. On the other hand, studies reporting the analysis of fatty acids (FA) from *E. huxleyi* showed elevated levels of *n*-3 PUFA, namely DHA (22:6*n*-3), stearidonic acid (SDA, 18:4*n*-3), and octadecapentaenoic acid (OPA, 18:5*n*-3) [10,12,13]. Unlike other microalgae, *E. huxleyi* contains small amounts of triacylglycerols as neutral lipids and instead contains alkenones, alkenoates, and alkenes [13]. As initially supported by thin-layer chromatography (TLC) data [13], PUFA present in *E. huxleyi* are mainly esterified in polar lipids. 

To date, the published work on the lipidome of *E. huxleyi* with characterization at the molecular level was focused on the changes in metabolism associated with viral infections, since the annual collapse of massive of blooms of *E. huxleyi* has been linked to viral control in the marine environment [14,15,16,17,18,19,20,21,22]. In addition, one of these studies evaluated the susceptibility to viral infection of diploid (2N) and haploid (1N) cells [21], since *E. huxleyi* has a biphasic life cycle consisting of diploid cells that produce coccoliths and uncalcified haploid cells. These studies were performed using liquid chromatography–mass spectrometry (LC–MS) with a reversed-phase C8 column [15,16,18,21,22], or a silica column with a diol function on C-chains [14,17,19,20,21]. Of all the lipid species identified, some studies only presented those that underwent significant changes after infection with the virus [18], or identified only glycosphingolipids, given their crucial role in the *E. huxleyi*–virus interaction [14,19,20]. Thus, the detailed identification of the composition of the polar lipidome of *E. huxleyi* at the molecular level still has a very relevant scientific goal. 

Hydrophilic interaction liquid chromatography (HILIC) is considered a variant of normal-phase chromatography and offers better separation of polar lipid classes than reversed-phase-based methods. The polar lipid profile at the molecular level of micro- [23] and macroalgae [24,25,26,27,28,29,30] has been disclosed using HILIC coupled with MS and tandem MS (MS/MS). Here, we present, for the first time, a detailed characterization of the polar lipidome of cultured *Emiliania huxleyi* RCC1250 (strain AC453), envisioning the scaling up of its cultivation (photobioreactors) to industrial volumes and its potential exploitation as a commercial source of high-valued lipids. The identification of polar lipid species was performed using high-resolution HILIC–MS and MS/MS and complemented by FA analysis by gas chromatography–mass spectrometry (GC–MS).

## 2. Materials and Methods 

### 2.1. Reagents

HPLC grade dichloromethane (CH_2_Cl_2_) and methanol (CH_3_OH) were purchased from Fisher Scientific Ltd. (Loughborough, UK). All other reagents were purchased from major commercial sources. Milli-Q water was obtained from a water purification system (Synergy, Millipore Corporation, Billerica, MA, USA). Phospholipid internal standards 1,2-dimyristoyl-*sn*-glycero-3-phosphocholine (dMPC), 1,2-dimyristoyl-*sn*-glycero-3-phosphoethanolamine (dMPE), 1,2-dimyristoyl-*sn*-glycero-3-phospho-(10-rac-glycerol) (dMPG), 1,2-dimyristoyl-*sn*-glycero-3-phospho-L-serine (dMPS), 1,2-dipalmitoyl-*sn*-glycero-3-phosphatidylinositol (dPPI), N-palmitoyl-D-*erythro*-sphingosylphosphorylcholine (NPSM), 1-nonadecanoyl-2-hydroxy-*sn*-glycero-3-phosphocholine (LPC (19:0)), 1,2-dimyristoyl-*sn*-glycero-3-propionic acid (dMPA), 1′,3′-bis[1–dimyristoyl-*sn*-glycero-3-phospho]-glycerol (tMCL) and N-heptadecanoyl-D-*erythro*-sphingosine (Cer (d18:1/17:0)) were purchased from Avanti Polar Lipids, Inc. (Alabaster, AL, USA).

### 2.2. Biomass Production

The strain of *Emiliania huxleyi* RCC1250 used was obtained from Roscoff Culture Collection (RCC; strain: AC453; origin: Alboran sea, Western Mediterranean). The starter inoculum was grown in 50 mL Erlenmeyer flasks in a climatic chamber (20 °C with 12:12 h light:dark cycle, using an irradiance of 50 µmol m^−2^ s^−1^) and the culture medium recommended by RCC, K/2 medium modified by Ian Probert [31]. Thereafter, the concentrated inoculum was scaled up to 500 mL and 5 L round flasks supplemented with NaHCO_3_ (0.087 g L^−1^) and modified algae medium [32] to a final nitrate concentration of 0.4 mM. All cultures were grown at the standard room temperature of the laboratory (22 ± 2 °C) under natural light without aeration, for about 15 days. All flasks were agitated manually, two times a day, to keep the cells in suspension. Cultures were monitored by optical density (750 nm) and microscopic observations. Samples were harvested during the late exponential growth phase by centrifugation (1735× *g* for 15 min) and freeze-dried (LyoQuest Telstar, Terrassa, Spain) for the proximal composition and lipidome analysis.

### 2.3. Moisture and Ash Determination

The residual moisture of freeze-dried biomass was determined by drying samples (50 mg × 3 replicates from a bulk sample) at 105 °C for 15 h in ceramic crucibles. After cooling to room temperature on a desiccator, the crucibles were then weighed to determine moisture by gravimetry. For the ash determination [33], the same biomass samples (previously dried at 105 °C) were incinerated in a muffle furnace at 575 °C for 6 h, cooled to room temperature, and weighed. In complement to this common method used in a muffle, the ash content was also determined by thermogravimetry (TG), using a protocol optimized for microalgae as follows: 5–10 mg of biomass submitted under an air atmosphere to a heating rate of 20 °C min^−1^ from room temperature to 600 °C (held for 30 min) [34].

### 2.4. Total Sugar Content Determination

*Emiliania huxleyi* biomass (2 mg × 3 replicates) was subjected to a prehydrolysis with 0.2 mL of 72% H_2_SO_4_ (*w*/*w*) for 3 h at room temperature, followed by 2.5 h hydrolysis with 1 M H_2_SO_4_ at 100 °C. Total sugar content was then determined by the phenol-sulfuric acid method [35]. For that, 1 mL of concentrated sulfuric acid was added to 80 µL of biomass hydrolysate in an ice bath, followed by addition of 150 µL of phenol solution (5%, *w*/*v*). Blanks were prepared without the addition of the phenol solution. After vigorous manual shaking, the tubes were placed in a bath at 100 °C for 10 min and then cooled in an ice bath. A calibration curve was prepared by performing the same procedure with glucose standards (0–0.6 mg mL^−1^). After new shaking, the absorbance of samples and standards was measured at 490 nm on a microplate UV-Vis spectrophotometer.

### 2.5. Neutral Sugars and Uronic Acids Analysis

*Emiliania huxleyi* biomass (2 mg × 3 replicates) was subjected to the same prehydrolysis and hydrolysis conditions described for phenol-sulfuric acid method. For determination of uronic acids (UA) content by m-phenylphenol colorimetric method [36], a volume of 500 µL of biomass hydrolysate was recovered after 1 h of hydrolysis at 100 °C with 1 M H_2_SO_4_ and diluted in 1 mL of distilled water. d-Galacturonic acid solutions (0–80 µg mL^−1^) were used to construct a calibration curve. To each tube containing 500 µL of sample/standard, 3 mL of 50 mM sodium borate prepared in concentrated sulfuric acid was added. After vigorously shaking, the tubes were placed in a bath at 100 °C for 10 min, followed by an ice bath. Then, 100 µL of MFF (m-phenylphenol 0.15% (*w*/*v*) in 0.5% (*w*/*v*) NaOH) was added to two of the three tubes of each sample and standard (replicates), followed by shaking and incubation in the dark for 30 min. The absorbance of each tube was measured at 520 nm after homogenization. 

For neutral sugars analysis, after hydrolysis at 100 °C for a total of 2.5 h, 200 µL of internal standard 2-desoxyglucose (0.1 mg mL^−1^) was added. Alditol acetates were prepared from 1 mL of each hydrolysate. After neutralization with 200 µL of NH_3_ 25%, alditol derivatives were obtained by reduction with 100 µL of NaBH_4_ (15% (*w*/*v*) in 3 M NH_3_) at 30 °C for 1 h. After cooling in an ice bath, the excess of borohydride was destroyed by the addition of glacial acetic acid (2 × 50 µL). The alditol derivatives (only 300 µL) were then acetylated with 3 mL of acetic anhydride in the presence of 450 µL of 1-methylimidazol at 30 °C for 30 min. To decompose the excess of acetic anhydride, distilled water (3 mL) was added with tubes in an ice bath. Alditol acetates were then extracted by adding 2.5 mL of dichloromethane. After vigorous manual shaking and centrifugation for 1 min at 1400× *g*, the aqueous phase was removed. An additional volume of dichloromethane (2.5 mL) and water (3 mL) was added, and the aqueous layer was removed using the same procedure. The organic phase was washed two times by the addition of distilled water (2 × 3 mL) and then evaporated to dryness. The dried material was dissolved in anhydrous acetone (2 × 1 mL), followed by the evaporation of acetone to dryness. Gas chromatography with a flame ionization detector (GC-FID) was used to identify alditol acetates [37]. The gas chromatograph (Perkin-Elmer Clarus 400) was equipped with a DB-225 capillary column (Agilent J&W GC columns, USA) with 30 m of length, 0.25 mm of internal diameter and 0.15 µm of film thickness. The injector temperature was 220 °C and the detector temperature was 230 °C. The oven was programmed for an initial temperature of 200 °C for 1 min, raised at 40 °C min^−1^ to 220 °C (held for 7 min), then raised at 20 °C min^−1^ to 230 °C (held for 1 min). Hydrogen was used as carrier gas at a 1.7 mL min^−1^ flow rate.

### 2.6. Nitrogen Determination and Protein Estimation

Nitrogen content of freeze-dried samples (2 mg × 3 replicates) was obtained by elemental analysis on a Leco Truspec-Micro CHNS 630-200-200 elemental analyzer at combustion furnace temperature 1075 °C and afterburner temperature 850 °C. Nitrogen was detected using thermal conductivity. The protein content was estimated from the nitrogen determination using 4.78 as nitrogen–protein conversion factor [38].

### 2.7. Lipid Extraction

Lipids were extracted using an adapted Folch method [23]. Microalgal biomass (20 mg × 3 replicates) was mixed with 2 mL of CH_2_Cl_2_:CH_3_OH (2:1) in a glass PYREX tube and homogenized by vortexing 2 min. After incubation at 30 °C for 30 min, the mixture was centrifuged (Selecta JP Mixtasel, Abrera, Barcelona, Spain) for 10 min at 626× *g* and the organic phase was collected in a new glass tube. The biomass residue was re-extracted twice with 2 mL of CH_2_Cl_2_:CH_3_OH (2:1) until a colorless pellet was obtained. The combined organic phases were dried under N_2_ stream.

To eliminate nonlipid contaminants, extracts were redissolved in 2 mL CH_2_Cl_2_ and 1 mL CH_3_OH. After vortexing for 1 min, 0.75 mL of Milli-Q water was added. The mixture was then vortexed for 1 min to allow mass transfer from the polar to nonpolar phase, followed by phase separation by centrifugation at 626× *g* for 10 min. The organic phase was collected in a new glass tube and the aqueous phase was re-extracted with 2 mL of CH_2_Cl_2_. The combined organic phases were then transferred to preweighed amber vials, dried under a stream of N_2_, weighed, and stored at −20 °C. Lipid content was estimated as a dry weight percentage.

### 2.8. Glycolipids and Phospholipids Quantification

Glycolipid quantification was achieved by the orcinol colorimetric method [39,40]. Briefly, 2 mL of orcinol solution (0.2% in 70% H_2_SO_4_) was added to 200 µg of each dried lipid extract (*n* = 3). After incubation at 80 °C for 20 min, samples were cooled to room temperature and absorbance was read at 505 nm. The sugar amount in the lipid extract was determined from a calibration curve prepared by performing the same reaction on known amounts of glucose (up to 40 μg, from an aqueous solution containing 5 mg mL^−1^ of sugar). The content of glycolipids was calculated by multiplying the amount of sugar by 2.8 [40,41].

Phospholipids were quantified by a molybdovanadate method [42], as routinely performed in the authors’ laboratory [25,43]. For that, 125 μL of 70% perchloric acid was added to 200 µg of each dried lipid extract (*n* = 3), previously transferred to a glass tube washed with 5% nitric acid. Samples were then incubated at 180 °C for 1 h on a heating block. After cooling to room temperature, 825 μL of Milli-Q water, 125 μL of ammonium molybdate (2.5 g 100 mL^−1^ in Milli-Q water), and 125 μL of ascorbic acid (0.1 g mL^−1^ in Milli-Q water) were added to each sample, with vortex mixing between each addition. Samples were incubated in a water bath at 100 °C for 10 min, and then immediately cooled down in a cold-water bath. Phosphate standards from 0.1 to 2 μg of phosphorus (P) were prepared from a sodium dihydrogen phosphate dihydrate (NaH_2_PO_4_·2H_2_O, 100 μg mL^−1^ of P), using the same experimental procedure as samples without the heating block step. The absorbance was measured at 797 nm. For each lipid extract, the amount of total phospholipid was calculated by multiplying the amount of P (determined by linear regression) by 25.

In both methods, the absorbance of standards and samples was measured on a microplate UV-Vis spectrophotometer (Multiskan GO, Thermo Scientific, Hudson, NH, USA).

### 2.9. Fatty Acid Analysis by Gas Chromatography–Mass Spectrometry (GC–MS)

Fatty acid methyl esters (FAMEs) were prepared by base-catalyzed transmethylation using 70 µg of each dried lipid extract (*n* = 3), followed by addition of 1 mL of internal standard (1.08 μg mL^−1^ of methyl nonadecanoate in n-hexane) and 200 μL of a methanolic solution of potassium hydroxide (2 M). After 2 min vortexing, 2 mL of an aqueous solution of sodium chloride (10 mg mL^−1^) was added and the sample was centrifuged for 5 min at 626× *g* [44]. The upper organic phase (600 µL) was collected and completely dried under a nitrogen stream. FAMEs were then redissolved in 30 µL of n-hexane and 3 μL was used for GC–MS analysis on a GC system (Agilent Technologies 6890 N Network, Santa Clara, CA, USA) equipped with a DB-FFAP column (30 m of length, 0.32 mm of internal diameter, and 0.25 µm of film thickness) (J&W Scientific, Folsom, CA, USA). The GC equipment was connected to an Agilent 5973 Network Mass Selective Detector. An electron impact mode was used at 70 eV, *m*/*z* range 50–550 and 1 s cycle in a full scan mode acquisition. 

The GC oven was programmed from an initial temperature of 80 °C for 3 min, followed by successive linear increases at 25 °C min^−1^ to 160 °C, at 2 °C min^−1^ to 210 °C, and 30 °C min^−1^ to 250 °C, standing at 250 °C for 10 min. The injector temperature was 220 °C, detector temperature was 280 °C, and helium (carrier gas) was used at a flow rate of 1.4 mL min^−1^. FA identification was based on retention times and MS spectra of FA standards (Supelco 37 Component FAME Mix, Sigma-Aldrich, Saint Louis, MI, USA), complemented with the analysis of MS spectra from Wiley 275 library and “The Lipid Web” [40]. FA quantification was performed using calibration curves obtained from FAME standards under the same instrumental conditions.

### 2.10. Hydrophilic Interaction Liquid Chromatography–Mass Spectrometry (HILIC–MS)

Lipid extracts were analyzed by hydrophilic interaction liquid chromatography (HILIC) on a high-performance LC (HPLC) system (Ultimate 3000 Dionex, Thermo Fisher Scientific, Bremen, Germany) with an autosampler coupled online to a Q-Exactive^®^ mass spectrometer with Orbitrap^®^ technology, as previously used for other algae [28,29,30]. Mobile phase A consisted of 25% water, 50% acetonitrile, and 25% methanol (per volume), with 2.5 mM ammonium acetate; mobile phase B consisted of 60% acetonitrile and 40% methanol, with the same amount of ammonium acetate in mobile phase A. Initially, 10% of mobile phase A was held isocratically for 2 min, followed by a linear increase to 90% of mobile phase A within 13 min and a maintenance period of 2 min, returning to the initial conditions in 8 min and maintained for 10 min. A volume of 10 µL of each sample, from a mixture containing 10 µL of lipid extract in CH_2_Cl_2_ (1 µg µL^−1^), 4 µL of a mixture of phospholipid standards (0.02 μg of dMPC, dMPE, NPSM, and LPC (19:0); 0.08 μg of dPPI, dMPA, and tMCL; 0.012 μg of dMPG; 0.04 μg of Cer (d18:1/17:0) and dMPS) and 86 µL of starting eluent, was introduced into the Ascentis Si column HPLC Pore column (10 cm × 1 mm, 3 µm, Sigma-Aldrich, St. Louis, MI, USA) with a flow rate of 50 µL min^−1^ at 35 °C. The mass spectrometer with Orbitrap^®^ technology was operated in simultaneous positive (electrospray voltage 3.0 kV) and negative (electrospray voltage −2.7 kV) modes with high resolution with 70,000 and AGC target of 1 × 10^6^, the capillary temperature was 250 °C, and the sheath gas flow was 15 U. In MS/MS experiments, a resolution of 17,500 and AGC target of 1 × 10^5^ was used and the cycles consisted in one full scan mass spectrum and 10 data-dependent MS/MS scans were repeated continuously throughout the experiments with the dynamic exclusion of 60 s and intensity threshold of 2 × 10^4^. Normalized collision energy™ (CE) ranged between 20, 25, and 30 eV. Data acquisition was performed using the Xcalibur data system (V3.3, Thermo Fisher Scientific, Bremen, Germany). 

LC–MS data processing was performed using MZmine 2 (version 2.32). Raw data was filtered for a RT from 0 to 30 min with m/z range of 400–1600 and a relative m/z tolerance of 5.0 ppm, a noise level of 1 × 10^4^ and typical retention time (RT) tolerance of 2 min were used. Chromatogram building was applied by means of the ADAP chromatogram builder module. The isotopic peaks grouper algorithm was applied with a maximum charge of 1 and the representative isotope was set to “lowest *m*/*z*”. Join aligner algorithm was set with weight for m/z 100 and weight for RT 10. Gap filling algorithm was also used with intensity tolerance of 100% and RT correction. The identification of the chromatographic peaks of interest was performed using in-house database (Supplementary Tables S1 and S2) created based on the information available on the LIPID MAPS. Duplicate peak filter algorithm with filter mode set to “new average” was applied. The peak identification was validated based on the typical RT of the respective lipid class and mass accuracy observed in LC–MS spectra (<5 ppm), as well as LC–MS/MS spectra interpretation that allowed to confirm the polar head group identity and the fatty acyl chains of the most molecular species.

## 3. Results

### 3.1. Biomass Composition

**Figure 1:**
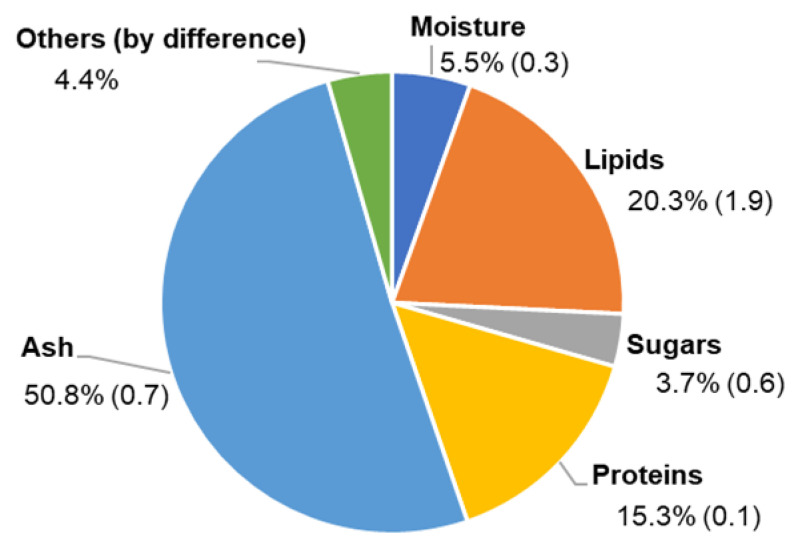
Composition of *Emiliania huxleyi* biomass (% of freeze-dried sample weight). Values are means of three replicates (standard deviations in parentheses).

The mean moisture content of *E. huxleyi* biomass was 5.5 ± 0.3%, while the ash content obtained by the traditional method at 575 °C in a muffle was 50.8 ± 0.7% (Figure 1). The ash content of *E. huxleyi* was also determined using TG at a temperature of 600 °C and a percentage of 48.0% was obtained.

Regarding the protein content, it was determined to be 15.3 ± 0.1% using 4.78 as the nitrogen–protein conversion factor (Figure 1). The sugar content determined according to the method of Dubois et al. [35] was 3.7 ± 0.1% (Figure 1). A similar total sugars content (3.3 ± 0.04%) was obtained by considering the neutral sugars quantified by GC-FID and the uronic acid content obtained by m-phenylphenol colourimetric method. Uronic acids (40.6 ± 6.8 mol%) were the most abundant sugars in *E. huxleyi*, followed by the neutral sugars galactose (16.4 ± 1.4 mol%), glucose (12.4 ± 1.2 mol%), mannose (8.6 ± 1.1 mol%), and rhamnose (7.8 ± 0.8 mol%). In minor amounts, arabinose (6.0 ± 1.0 mol%), fucose (4.9 ± 0.6 mol%), and xylose (1.5 ± 0.3 mol%) were also identified.

*E. huxleyi* revealed the presence of 20.3 ± 2.0% lipid content (Figure 1). In a first approach of the polar lipid composition in the total lipid extracts, the glycolipids and the phospholipids were quantified by colorimetric assays. The contents of glycolipids were 84.9 ± 6.4 µg mg^−1^ extract and phospholipids was 19.1 ± 4.5 µg mg^−1^ extract.

### 3.2. Fatty Acid Profile

The determination of the fatty acid composition of *E. huxleyi* by GC–MS showed the presence of 43.6 ± 2.5% saturated FA (SFA), 16.4 ± 0.9% monounsaturated FA (MUFA), and 40.0 ± 3.2% PUFA (Table 1). The predominant SFA detected were 16:0 (17.1 ± 1.8%), 18:0 (13.4 ± 1.6%), and 14:0 (9.2 ± 1.4%). The most abundant MUFA were 18:1*n*-7 (7.7 ± 0.3%) and 18:1*n*-9 (6.6 ± 0.2%). In terms of PUFA, only the *n*-3 PUFA was identified and the major ones were 22:6*n*-3 (DHA, 17.2 ± 2.7%), 18:4*n*-3 (SDA, 11.0 ± 0.4%), and 18:5*n*-3 (6.6 ± 0.5%).

### 3.3. Identification of Polar Lipids by LC–MS

**Figure 2:**
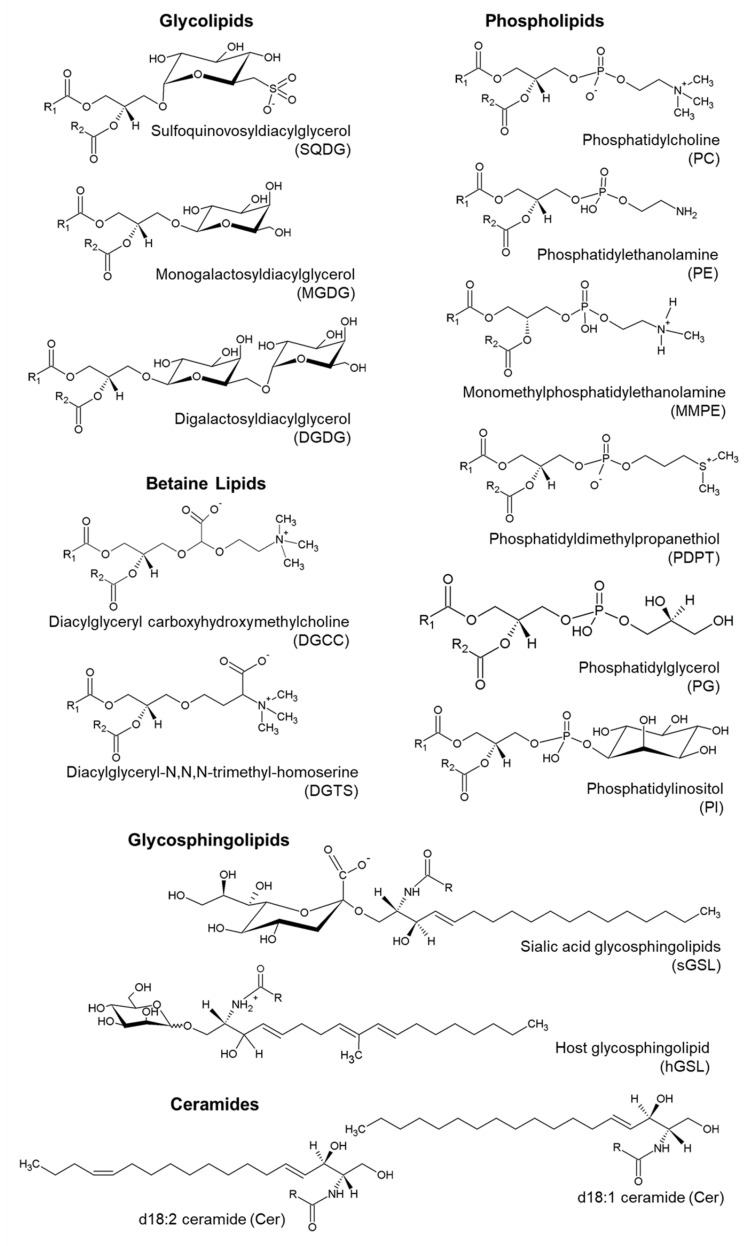
Representative structures of the different classes of lipids identified in *E. huxleyi*. Different species identified in the same class have distinct fatty acyl chains, represented by R_1_ and R_2_ for the diacyl-forms. The lyso forms have a single fatty acyl chain.

*Emiliania huxleyi* polar lipidome was characterized by HILIC–MS and HILIC–MS/MS (referred for simplicity as LC–MS and LC–MS/MS) (Supplementary Figure S1). Overall, 134 lipid species were identified, categorized by classes of glycerolipids (glycolipids, betaine lipids, and phospholipids), and sphingolipids (ceramides and glycosphingolipids) (Figure 2).

#### 3.3.1. Glycerolipids

##### Glycolipids

The glycolipids identified were distributed into two classes of acidic sulfolipids, sulfoquinovosyldiacylglycerol (SQDG) and sulfoquinovosylmonoacylglycerol (SQMG), and into four classes of neutral galactolipids, digalactosyldiacylglycerol (DGDG), monogalactosyldiacylglycerol (MGDG), digalactosylmonoacylglycerol (DGMG), and monogalactosylmonoacylglycerol (MGMG).

Acidic sulfolipids were observed by LC–MS in negative ion mode as [M−H]^−^ ions (Table 2). The most abundant SQDG (28:0) was assigned at *m*/*z* 737.4534, followed by SQDG (30:0) at *m*/*z* 765.4843 and SQDG (36:7) at *m*/*z* 835.4692. SQMG (14:0), identified at *m*/*z* 527.2540, was the most abundant of this class (Supplementary Figure S2). The identification of the sulfolipid species was confirmed by a typical product ion at *m*/*z* 225.0061, corresponding to the anion of the sulfoquinovosyl polar head group (C_6_H_9_O_4_SO_3_^−^), observed in MS/MS spectra of [M–H]^–^ ions of both SQMG and SQDG species. The product ions resulting from neutral losses of fatty acyl chains as carboxylic acid (RCOOH) allowed the identification of the fatty acyl composition. As the lyso forms have only one FA, the MS/MS spectra of SQMG species showed a single neutral loss of RCOOH [27,44,45]. As an example, the LC–MS/MS spectrum of SQDG (30:0) is shown in Supplementary Figure S2.

Neutral glycolipids were observed in positive LC–MS spectra as [M + NH_4_]^+^ ions (Table 3). The most abundant MGDG was detected at *m*/*z* 784.5000, corresponding to MGDG (36:10). DGDG (36:10) at *m*/*z* 946.5539 was the most abundant DGDG, followed by DGDG (28:0) at *m*/*z* 854.5859 and DGDG (32:1) at *m*/*z* 908.6323 (Supplementary Figure S2).

The identification of neutral glycolipids was confirmed by the typical fragmentation observed in the LC–MS/MS spectra of the respective [M+NH_4_]^+^ ions. MGMG and MGDG species were assigned by the typical neutral loss resultant from the combined neutral loss of NH_3_ and galactosyl unit (−197 Da), while DGDG and DGMG species were identified by the typical neutral loss resultant from the combined neutral loss of NH_3_ and digalactosyl unit (−359 Da). The fatty acyl chains were identified by the presence of product ions corresponding to each fatty acyl group as an acylium ion plus 74 Da of the glycerol backbone (RCO + 74 Da) [46]. A representative LC–MS/MS spectrum of the DGDG class is shown in Supplementary Figure S4.

##### Betaine Lipids

The betaine lipids identified in this study included diacylglyceryl-N,N,N-trimethyl-homoserine (DGTS), monoacylglyceryl-N,N,N-trimethyl-homoserine (MGTS), and diacylglyceryl carboxyhydroxymethylcholine (DGCC). Among these classes of betaine lipids, DGCC is the least reported in microalgae, in which its occurrence has been associated with the phylum Haptophyta [47,48]. Additionally, lipid species of unknown structure, previously reported in *E. huxleyi* and classified based on exact mass, predicted formula and MS/MS as betaine-like lipids (BLL), due to lack of phosphate and the presence of reduced nitrogen in the headgroup [17], were identified in this study.

Betaine lipids were observed in positive LC–MS spectra as [M + H]^+^ ions (Table 4). DGTS (32:1), detected at *m*/*z* 710.5940, and MGTS (14:0), detected at *m*/*z* 446.3483, were the most abundant of these classes. The major DGCC (36:6) was observed at *m*/*z* 772.5738, while the most abundant BLL (38:6) was identified at *m*/*z* 830.5798 (Supplementary Figure S5). 

An LC–MS/MS spectrum representative of DGTS is shown in Supplementary Figure S6. This MS/MS spectrum showed the typical product ion of DGTS and MGTS at *m*/*z* 236.1559 (C_10_H_22_O_5_N^+^), resulting from the combined loss of the two fatty acids as ketene derivatives (R_1_CO+R_2_CO) [44,45,49]. The fatty acyl composition was found by identifying the neutral losses of fatty acyl chains as acid (-RCOOH) and ketene (-R=C=O) derivatives.

As shown for DGCC (36:6) (Supplementary Figure S7a), the fragmentation under LC–MS/MS conditions of the [M + H]^+^ ions of DGCC gave rise to product ions at *m*/*z* 104.1072 (C_5_H_14_NO^+^) and *m*/*z* 132.1017 (C_6_H_14_NO_2_^+^), corroborating the identification of the DGCC polar head group [47,50]. Fatty acyl composition of DGCC was identified from fatty acyl carboxylate anions (RCOO^–^) observed in the MS/MS of [M+CH_3_COO]^–^ ions (Supplementary Figure S7b).

As previously observed in MS/MS data from ion-trap and FT-ICR instruments [17], the fragmentation of [M + H]^+^ ions from BLL under Orbitrap-based LC–MS/MS conditions gave rise to a product ion at *m*/*z* 190.0704 (C_7_H_12_O_5_N) (Supplementary Figure S8).

##### Phospholipids

The phospholipids identified in this study included phosphatidylcholine (PC), phosphatidylethanolamine (PE), monomethylphosphatidylethanolamine (MMPE) phosphatidyldimethylpropanethiol (PDPT), phosphatidylinositol (PI), phosphatidylglycerol (PG), and lyso forms (LPC and LPE). PC, LPC, PE, LPE, MMPE, and PDPT species were identified in the positive mode as [M + H]^+^ ions (Table 5). The most abundant PC were identified as PC (30:3), PC (36:2), and PC (36:6) at *m*/*z* 700.4909, 786.6019, and 778.5397, respectively. With respect to PE, the most abundant ones identified were PE (30:3) at *m*/*z* 658.4435 and PE (36:2) at *m*/*z* 744.5553. The most abundant PDPT was PDPT (36:6) at *m*/*z* 795.5009 (Supplementary Figure S9).

In terms of LC–MS/MS spectra of [M + H]^+^ ions, PC and LPC species showed the typical product ion at *m*/*z* 184.0737, corresponding to the phosphocholine polar head (Supplementary Figure S10a) [46], while PE and LPE species showed the characteristic neutral loss of 141 Da (C_2_H_8_NO_4_P) (Supplementary Figure S11a). The fatty acyl composition was determined by LC–MS/MS analysis of the [M + CH_3_COO]^−^ ions in case of PC and LPC, and [M – H]^–^ ions in the case of PE and LPE, showing the presence of fatty acyl carboxylate anions (RCOO^–^) (Supplementary Figures S10b and S11b).

In this study, for the first time, a species of MMPE was identified in *E. huxleyi*. A previous study reported the presence of dimethylphosphatidylethanolamines (DMPE) in *E. huxleyi* [18], but not of MMPE. The MMPE identified was MMPE (30:1), which is an isomer of PE (31:1). These isomeric species were discriminated because they eluted in different retention times (Supplementary Figure S12a) and their MS/MS spectra showed distinct neutral losses. Distinctly from the [M + H]^+^ ions of PE which showed the neutral loss of 141 Da, MMPE (30:1) showed the neutral loss of 155 Da (C_3_H_10_NO_4_P), corresponding to the MMPE polar head group (Supplementary Figure S12b,c) [51]. The MMPE (30:1) species was also identified in negative mode as [M – H]^–^ ions, based on retention time and exact mass, but the respective MS/MS was not acquired due to its low abundance.

As shown for PDPT (36:6) (Supplementary Figure S13), the fragmentation of [M + H]^+^ ions from PDPT under LC–MS/MS conditions yielded a product ion at *m*/*z* 201.0340 (C_5_H_14_O_4_PS^+^), corresponding to the PDPT polar head [17]. In negative mode, the PDPT lipid species were observed as [M – CH_3_COO]^–^ ions, and not as [M – CH_3_ + CH_3_COO]^–^, [M – CH_3_] ^–^ and [M – H – S(CH_3_)_2_]^–^ ions previously reported from ion-trap MS data [52]. Like PC, fatty acyl carboxylate anions (RCOO^–^) could be expected in the LC–MS/MS spectra of [M – CH_3_COO]^–^ ions of PDPT (data not available).

The PG and PI species were observed in LC–MS as [M – H]^–^ ions. Overall, 11 PG and 2 PI species were identified (Table 6). The most abundant PG species were assigned as PG (34:1) at *m*/*z* 747.5200 and PG (36:2) at *m*/*z* 773.5355. PI species were present in lower numbers, with the PI (38:6) at *m*/*z* 881.5196 being the most abundant (Supplementary Figure S9).

The LC–MS/MS spectra of [M – H]^–^ ions of PG species (Supplementary Figure S14) showed the presence of the product ion at *m*/*z* 171.0063 (C_3_H_7_O_2_OPO_3_H), corresponding to the glycerol phosphate anion. On the other hand, the polar head of PI (Supplementary Figure S15) was corroborated by the presence of the product ion at *m*/*z* 241.0115 (C_6_H_10_O_5_PO_3_), corresponding to an inositol-1,2-cyclic phosphate anion. The carboxylate anions (RCOO^–^) allowed the identification of fatty acyl chains [46].

#### 3.3.2. Sphingolipids

##### Glycosphingolipids

The glycosphingolipids identified in *E huxleyi* lipidome included sialic acid glycosphingolipids (sGSL) and host GSL (hGSL). Glycosphingolipids were observed in positive LC–MS spectra as [M + H]^+^ ions (Table 7, Supplementary Figure S16).

In negative mode, sGSL were observed as [M − H]^−^ ions. The LC–MS/MS spectrum of [M − H]^−^ ions of sGSL (d40:2) showed the product ion at *m*/*z* 249.0617 (C_9_H_13_O_8_) (Supplementary Figure S17a), corroborating the presence of a sialic acid 2-keto-3-deoxynononic acid (Kdn) in the sGSL head group [17]. Additionally, two product ions were observed at *m*/*z* 129.0180 (C_5_H_5_O_4_) and 87.0075 (C_3_H_3_O_3_), resulting from cross-ring cleavages of the Kdn. Sphingosine d18:1 was confirmed by the LC–MS/MS spectrum of the respective [M + H]^+^ ions, showing the presence of product ions at *m*/*z* 264.2673 and 282.2737. This spectrum also showed the product ion at *m*/*z* 604.5978, resulting from a neutral loss of Kdn. The fatty acyl composition was found by the mass difference (322 Da) between product ions at *m*/*z* 604.5978 and 282.2737, corresponding to the fatty acid 22:0 as a ketene derivative (Supplementary Figure S17b).

The LC–MS/MS spectrum of hGSL at *m*/*z* 806.6159 (Supplementary Figure S18) showed product ions at *m*/*z* 626.5516 (−180 Da) and 608.5402 (−198 Da), resulting from glycosidic cleavage with neutral loss of a hexose and an additional loss of water, respectively. Additionally, an amino fatty acid product ion (*m*/*z* 334.3097), and long-chain base product ions (*m*/*z* 275.2365 and 257.2260) were observed. These were similar to the fragmentation pathways previously observed in ion-trap MS/MS data [19].

##### Ceramides

In this study, ceramides were identified in positive LC–MS spectra as [M + H]^+^ ions. The most abundant ceramides were assigned as Cer(d38:2) at *m*/*z* 592.5672 and Cer(d40:1) at *m*/*z* 622.6143 (Supplementary Figure S16). The species of this class were confirmed by LC–MS/MS; for example, in the spectrum of Cer(d40:2) it is possible to identify the sphingodienine ion at *m*/*z* 262.2525 (Supplementary Figure S19).

## 4. Discussion

*Emiliania huxleyi* biomass was analyzed for the content (expressed as a percentage of freeze-dried sample weight) of moisture and ash, protein, sugar, and lipids. In terms of ash content, two methods were used: the traditional method at 575 °C in a muffle and TG at a temperature of 600 °C, which has been proposed as a more reasonable terminal temperature for the determining the ash content of microalgae [34]. A similar content was obtained for the two methods (48.0% by TG versus 50.8% by muffle furnace) with the advantages of the TG method, which requires only a few milligrams of biomass and is more automated than the muffle method. The high ash content of cultured *E. huxleyi* when compared with non-coccolithophore microalgae [53] can be due to the presence of calcium carbonate, as its decomposition is expected above 600 °C [54].

Protein content was determined as 15.3% with 4.78 as nitrogen-protein conversion factor. This factor was proposed for marine microalgae, as the traditional factor 6.25 could lead to an overestimation of the protein content in the biomass of microalgae [38].

Regarding sugar content, a similar total sugars content was obtained with different methods. Lipids content was determined as 20.3%, which is within the range of those previously reported for other haptophytes harvested at the late exponential growth phase (25.5 ± 2.4% for *Pseudoisochrysis paradoxa* and 24.3 ± 3.8% for *Diacronema vlkianum*) [48].

Together, the contents of glycolipids (84.9 ± 6.4 µg mg^−1^ extract) and phospholipids (19.1 ± 4.5 µg mg^−1^ extract) estimated by colorimetry represented approximately 10% of the total lipid extract. Indeed, other classes of polar lipids, in addition to glycolipids and phospholipids, were previously identified in *E. huxleyi* [15,18,22], namely betaine lipids which are not quantified by the colorimetric methods used. Polar lipids were previously estimated (by TLC) to represent approximately 50% of the total lipids extracted from eight isolates of *E. huxleyi* with chloroform:methanol (2:1, *v*/*v*), in logarithmic or stationary phases [13]. A similar content of polar lipids (also determined by TLC) was reported for other haptophytes (49.4% for *P. paradoxa* and 61.8% for *D. vlkianum*) [48]. On the other hand, the lipid extract can contain significant amounts of pigments (corroborated by the green colour of the extract), as well as sterols and long-chain alkenones, alkenoates, and alkenes [13]. Although neutral lipids are not the subject of the present study, these compounds may also be important from the perspective of potential applications. For example, alkenones from haptophytes have been reported as promising renewable phase change materials [55].

The fatty acid analysis revealed the presence of high levels of *n*-3 PUFA, namely 22:6*n*-3 (DHA, 17.2 ± 2.7%), 18:4*n*-3 (SDA, 11.0 ± 0.4%), and 18:5*n*-3 (6.6 ± 0.5%), which is in agreement with what has been previously described in literature [13,56,57,58,59]. This profile gives *E. huxleyi* increased potential as a source of these compounds, which have been described with significant activity in reducing cardiovascular disease, morbidity, and mortality [60,61,62,63,64], as well as visual and neurological development and improvement in inflammatory conditions, such as arthritis and asthma [65,66,67].

All classes of polar lipids identified in this study, except monomethylphosphatidylethanolamine (MMPE), have been reported in previous studies with other strains of *E. huxleyi* [15,18,22]. The lipid species identified in each class are not the same as those previously reported [18,22], which may be due to the strain and growth conditions of the microalgae, as well as to the different experimental conditions used for the extraction and analysis of lipids. Some fatty acids identified as components of the polar lipids were not seen in the fatty acid analysis by GC–MS, due to higher sensitivity of LC–MS compared to GC–MS.

In terms of bioactivity of sulfolipids, it should be noted that SQDG (16:0/16:0), also present in *E. huxleyi*, was previously described as a lipid with antimicrobial activity [68]. SQDG (20:5/16:0), also identified in *E. huxleyi*, was described with antimicrobial [69] and anti-inflammatory effects [70], which reinforces the potential use of this microalga as a source of bioactive lipids with potential health benefits. Neutral glycolipids from microalgae were also previously reported with bioactive properties, namely MGDG from *Nannochloropsis* sp. showed anti-inflammatory activity and MGDG from *Chlorella vulgaris* exhibited antitumor activity [8,71].

Betaine lipids have also been identified in several classes of microalgae [29,72] and some studies have been developed to identify their biological activities and possible applications. In particular, DGTS species isolated from the microalgae *Nannochloropsis granulata* were described as potential anti-inflammatory agents, exhibiting anti-inflammatory activity by inhibiting nitric oxide (NO) production in RAW264.7 macrophage cells with downregulation of inducible nitric oxide synthase expression [73]. Given the presence of betaine lipids in the biomass of *E. huxleyi*, it could be a future challenge to investigate its application as a source of these bioactive compounds

From the point of view of relevance for biotechnological applications, phospholipids were also considered to be important bioactive compounds, namely anti-inflammatory PG were isolated from the red macroalga *Palmaria palmata*, exhibiting strong and dose-dependent NO inhibitory activity [70].

In respect of glycosphingolipids, sGSL were recently described as indicative of susceptibility to viral infection in *E. huxleyi* [17], and another class previously classified as host GSL (hGSL) due to its prevalence in uninfected *E. huxleyi* [14,19]. Viral GSL, previously described as potential biomarkers for viral infection [14], were not identified in this study with cultured *E. huxleyi*.

Previous studies have identified ceramides as highly bioactive compounds with significant effects on cell metabolism [74,75]. Wertz and his collaborators [76] investigated the uptake of several sphingoid bases by *Escherichia coli* and *Staphylococcus aureus*, and assessed subsequent ultrastructural damage, exploiting the potential for prophylactic or therapeutic purposes. Ceramides with antibacterial activity were also reported against pathogenic Neisseria [77]. Considering the complexity of the biological processes affected by this category of compounds [75], further work is still needed to establish the relevance of *E. huxleyi* sphingolipids for potential biotechnological applications.

## 5. Conclusions

In this study, the polar lipidome profile of the coccolithophore *Emiliania huxleyi* (strain AC453) was described in detail using HILIC–MS and GC–MS. *E. huxleyi* revealed the presence of distinct beneficial *n*-3 PUFA, representing about 40% of total FA. Their high amount underlines the importance of this microalga as a natural source of *n*-3 PUFA, in particular, 22:6*n*-3 (DHA) and 18:4*n*-3 (SDA), which are naturally esterified in various polar lipids. Among the classes of polar lipids identified in the biomass of *E. huxleyi*, glycerolipids, especially glycolipids, are the most recognized as bioactive compounds and linked to health benefits. Thus, the polar lipids of *E. huxleyi* show great potential for future biorefinery approaches, envisioning their application in the development of new products and materials, especially as ingredients in food and feed products, drugs and cosmetics.

## Figures and Tables

**Table 1:** Fatty acid (FA) composition of *E. huxleyi* determined by gas chromatography–mass spectrometry (GC–MS) analysis of fatty acid methyl ester derivatives. Values are means ± standard deviation (SD) of the different lipid extracts obtained from a bulk sample (*n* = 3). SFA, saturated fatty acid; MUFA, monounsaturated fatty acid; PUFA, polyunsaturated fatty acid.

Fatty Acids (% of Total FA)	Amount (±SD, *n* = 3)
14:0	9.2 ± 1.4
13-methyl-14:0 (iso)	2.4 ± 0.1
15:0	1.5 ± 0.2
16:0	17.1 ± 1.8
16:1*n*-9	0.7 ± 0.1
16:1*n*-7	1.4 ± 0.4
18:0	13.4 ± 1.6
18:1*n*-9	6.6 ± 0.2
18:1*n*-7	7.7 ± 0.3
18:3*n*-3	5.3 ± 0.2
18:4*n*-3	11.0 ± 0.4
18:5*n*-3	6.6 ± 0.5
22:6*n*-3	17.2 ± 2.7
Σ SFA	43.6 ± 2.5
Σ MUFA	16.4 ± 0.9
Σ PUFA	40.0 ± 3.2
Total FA (µg mg^−1^ extract)	85.1 ± 12.5

**Table 2:** Sulfolipid species identified by liquid chromatography–mass spectrometry (LC–MS) in negative mode as [M-H]^−^ ions (error < 5 ppm). The identification as a sulfolipid and fatty acyl composition was confirmed by analysis of the tandem MS (LC–MS/MS) spectra of [M–H]^–^ ions. The numbers in parentheses (C:N) indicate the total number of carbon atoms (C) and double bonds (N) in fatty acyl chains. The most abundant species of each class (relative abundance > 10 %) are indicated in bold. SQMG, sulfoquinovosylmonoacylglycerol; SQDG, sulfoquinovosyldiacylglycerol.

Lipid Species (C:N)	Observed *m*/*z*	Theoretical *m*/*z*	Error (ppm)	Fatty Acyl Chain (s)	Formula
SQMG (14:0)	527.2540	527.25261	2.6321	14:0	C_23_H_43_O_11_S
SQMG (18:4)	575.2541	575.25261	2.5218	18:4	C_27_H_43_O_11_S
SQMG (22:6)	627.2859	627.28391	3.0996	22:6	C_31_H_47_O_11_S
**SQDG (28:0)**	737.4534	737.4510	3.2395	14:0-14:0	C_37_H_69_O_12_S
**SQDG (30:0)**	765.4843	765.4823	2.5904	14:0-16:0	C_39_H_73_O_12_S
SQDG (30:1)	763.4668	763.4666	0.2376	14:0-16:1, 14:1-16:0	C_39_H_71_O_12_S
SQDG (32:0)	793.5124	793.5136	−1.5073	16:0-16:0, 18:0-14:0	C_41_H_77_O_12_S
SQDG (32:1)	791.5006	791.4979	3.3639	14:0-18:1, 16:1-16:0	C_41_H_75_O_12_S
SQDG (32:2)	789.4825	789.4823	0.3421	14:0-18:2	C_41_H_73_O_12_S
SQDG (32:3)	787.4688	787.4666	2.8122	14:0-18:3	C_41_H_71_O_12_S
SQDG (32:4)	785.4545	785.4510	4.4448	14:0-18:4	C_41_H_69_O_12_S
SQDG (32:5)	783.4360	783.4353	0.8766	14:0-18:5	C_41_H_67_O_12_S
SQDG (34:1)	819.5288	819.5292	−0.4555	16:0-18:1, 17:0-17:1	C_43_H_79_O_12_S
SQDG (34:4)	813.4846	813.4823	2.8541	16:0-18:4	C_43_H_73_O_12_S
SQDG (34:6)	809.4518	809.4510	1.0215	16:2-18:4	C_43_H_69_O_12_S
SQDG (36:1)	847.5594	847.5605	−1.3057	22:1-14:0; 20:1-16:0	C_45_H_83_O_12_S
SQDG (36:2)	845.5466	845.5449	2.0742	16:0-20:2, 18:1-18:1	C_45_H_81_O_12_S
SQDG (36:5)	839.5003	839.4979	2.7927	18:1-18:4, 20:5-16:0, 22:5-14:0	C_45_H_75_O_12_S
SQDG (36:6)	837.4813	837.4823	−1.2034	18:4-18:2, 14:0-22:6, 18:3-18:3	C_45_H_73_O_12_S
**SQDG (36:7)**	835.4692	835.4666	3.0794	18:3-18:4	C_45_H_71_O_12_S
SQDG (36:8)	833.4516	833.4510	0.7304	18:4-18:4	C_45_H_69_O_12_S
SQDG (36:9)	831.4378	831.4353	3.0652	18:4-18:5	C_45_H_67_O_12_S
SQDG (38:2)	873.5791	873.5761	3.4656	18:1-20:1	C_47_H_85_O_12_S
SQDG (38:6)	865.5158	865.5136	2.5672	16:0-22:6, 18:4-20:2, 18:3-20:3	C_47_H_77_O_12_S
SQDG (38:9)	859.4697	859.4666	3.5523	20:5-18:4	C_47_H_71_O_12_S
SQDG (40:10)	885.4853	885.4822	3.5017	18:4-22:6	C_49_H_73_O_12_S
SQDG (44:12)	937.5164	937.5136	3.0468	22:6-22:6	C_53_H_77_O_12_S

**Table 3:** Galactolipid species identified by liquid chromatography–mass spectrometry (LC–MS) in positive mode as [M + NH_4_]^+^ ions (error < 5 ppm). The identification as a galactoglycerolipids and fatty acyl composition was confirmed by analysis of tandem MS (LC–MS/MS) spectra of [M+NH_4_]^+^ ions. The numbers in parentheses (C:N) indicate the number of carbon atoms (C) and double bonds (N) in fatty acyl chains. The most abundant species of each class (relative abundance > 10%) are indicated in bold. Species identified only by retention time and mass accuracy are marked with an asterisk (*). MGMG, monogalactosylmonoacylglycerol; MGDG, monogalactosyldiacylglycerol; DGMG, digalactosylmonoacylglycerol; DGDG, digalactosyldiacylglycerol.

Lipid Species (C:N)	Observed *m*/*z*	Theoretical *m*/*z*	Error (ppm)	Fatty Acyl Chain (s)	Formula
MGMG (14:0)	482.3330	482.3329	0.1768	14:0	C_23_H_48_NO_9_
MGMG (16:0)	510.3636	510.3642	−1.1756	*	C_25_H_52_NO_9_
MGMG (18:1)	536.3799	536.3799	0.1570	*	C_27_H_54_NO_9_
MGMG (18:3)	532.3497	532.3486	2.0900	*	C_27_H_50_NO_9_
MGMG (18:4)	530.3341	530.3329	2.2083	18:4	C_27_H_48_NO_9_
**MGMG (18:5)**	528.3175	528.3173	0.5232	*	C_27_H_46_NO_9_
MGMG (20:5)	556.3487	556.3486	0.2706	*	C_29_H_50_NO_9_
MGMG (22:6)	582.3648	582.3642	1.0647	*	C_31_H_52_NO_9_
MGDG (28:0)	692.5326	692.5307	2.7298	*	C_37_H_74_NO_10_
MGDG (32:1)	746.5792	746.5777	2.0069	14:0-18:1	C_41_H_80_NO_10_
MGDG (32:3)	742.5439	742.5464	−3.4143	*	C_41_H_76_NO_10_
MGDG (32:4)	740.5320	740.5307	1.8200	*	C_41_H_74_NO_10_
MGDG (34:1)	774.6084	774.6090	−0.7271	*	C_43_H_84_NO_10_
MGDG (36:2)	800.6249	800.6252	−0.3670	*	C_45_H_8_6NO_10_
MGDG (36:4)	796.5967	796.5933	4.2891	*	C_45_H_82_NO_10_
MGDG (36:8)	788.5304	788.5313	−1.0574	18:4-18:4	C_45_H_74_NO_10_
MGDG (36:9)	786.5152	786.5156	−0.4468	*	C_45_H_72_NO_10_
**MGDG (36:10)**	784.5000	784.5000	0.0065	18:5-18:5	C_45_H_70_NO_10_
MGDG (38:5)	822.6092	822.6095	−0.3692	*	C_47_H_84_NO_10_
**MGDG (38:6)**	820.5947	820.5939	0.9627	*	C_47_H_82_NO_10_
**MGDG (38:9)**	814.5452	814.5469	−2.0729	*	C_47_H_76_NO_10_
MGDG (40:10)	840.5633	840.5626	0.9151	*	C_49_H_78_NO_10_
MGDG (40:11)	838.5491	838.5469	2.6480	*	C_49_H_76_NO_10_
MGDG (40:3)	854.6719	854.6721	−0.2641	*	C_49_H_92_NO_10_
MGDG (44:12)	892.5956	892.5939	1.9808	*	C_53_H_82_NO_10_
**DGMG (18:5)**	690.3709	690.3701	1.1385	*	C_33_H_56_NO_14_
**DGDG (28:0)**	854.5859	854.5841	2.1204	*	C_43_H_84_NO_15_
**DGDG (32:1)**	908.6323	908.6310	1.4458	18:1-14:0	C_47_H_90_NO_15_
DGDG (36:5)	956.6292	956.6310	−1.8642	*	C_51_H_90_NO_15_
DGDG (36:6)	954.6145	954.6154	−0.9079	*	C_51_H_88_NO_15_
DGDG (36:8)	950.5829	950.5841	−1.2570	*	C_51_H_84_NO_15_
**DGDG (36:10)**	946.5539	946.5528	1.1713	18:5-18:5	C_51_H_80_NO_15_
DGDG (40:11)	1000.6029	1000.5997	3.2294	*	C_55_H_86_NO_15_

**Table 4:** Betaine lipid species identified by liquid chromatography–mass spectrometry (LC–MS) in positive mode as [M + H]^+^ ions (error < 5 ppm). The identification as betaine lipids and fatty acyl composition of DGTS and MGTS was confirmed by analysis of tandem MS (LC–MS/MS) spectra of [M + H]^+^ ions. The fatty acyl composition of DGCC was determined by LC–MS/MS spectra of [M + CH_3_COO]^–^ ions. The numbers in parentheses (C:N) indicate the number of carbon atoms (C) and double bonds (N) in fatty acyl chains. The most abundant species of each class (relative abundance > 10%) are indicated in bold. Species identified only by retention time and mass accuracy are marked with an asterisk (*). Species identified by retention time, mass accuracy, and confirmation of polar head by positive LC–MS/MS are marked with two asterisks (**). MGTS, monoacylglyceryl-N,N,N-trimethyl-homoserine; DGTS, diacylglyceryl-N,N,N-trimethyl-homoserine; DGCC, diacylglyceryl carboxyhydroxymethylcholine; BLL, betaine like lipids.

Lipid Species (C:N)	Observed *m*/*z*	Theoretical *m*/*z*	Error (ppm)	Fatty Acyl Chain (s)	Formula
MGTS (14:0)	446.3483	446.3482	0.3670	14:0	C_24_H_48_O_6_N
MGTS (20:5)	520.3645	520.3638	1.3522	20:5	C_30_H_50_O_6_N
**DGTS (30:0)**	684.5791	684.5778	1.9458	15:0-15:0; 14:0-16:0	C_40_H_78_O_7_N
**DGTS (32:1)**	710.5940	710.5935	0.6954	18:1-14:0	C_42_H_80_O_7_N
DGTS (32:2)	708.5776	708.5778	−0.2396	16:1-16:1	C_42_H_78_O_7_N
DGTS (32:3)	706.5613	706.5622	−1.3216	*	C_42_H_76_O_7_N
**DGTS (32:7)**	698.5001	698.4996	0.7037	*	C_42_H_68_O_7_N
DGTS (34:1)	738.6252	738.6248	0.5559	16:0-18:1	C_44_H_84_O_7_N
DGTS (34:4)	732.5780	732.5778	0.2284	*	C_44_H_78_O_7_N
**DGTS (36:2)**	764.6416	764.6404	1.6039	18:1-18:1	C_46_H_86_O_7_N
**DGCC (36:6)**	772.5738	772.5727	1.4238	22:6-14:0	C_46_H_78_O_8_N
DGCC (40:7)	826.6216	826.6197	2.2985	**	C_50_H_84_O_8_N
**DGCC (44:12)**	872.6045	872.6040	0.5730	**	C_54_H_82_O_8_N
**BLL (38:6)**	830.5798	830.5782	1.9264	**	C_48_H_80_O_10_N
**BLL (40:7)**	856.5946	856.5939	0.8172	**	C_50_H_82_O_10_N

**Table 5:** Phospholipid species identified by liquid chromatography–mass spectrometry (LC–MS) in positive mode as [M + H]^+^ ions. Polar head groups were confirmed by tandem MS (LC–MS/MS) spectra of [M + H]^+^ ions. The fatty acyl composition of PC was determined by LC–MS/MS spectra of [M + CH_3_COO]^–^ ions, while the composition of PE was determined by LC–MS/MS of [M – H]^–^ ions. The numbers in parentheses (C:N) indicate the number of carbon atoms (C) and double bonds (N) in fatty acyl chains. The most abundant species (relative abundance > 10%) are indicated in bold. Species identified only by retention time and mass accuracy are marked with an asterisk (*). Species identified by retention time, mass accuracy, and confirmation of polar head by positive MS/MS are marked with two asterisks (**). Isomeric species are marked with a symbol (†). PC, phosphatidylcholine; LPC, lysophosphatidylcholine; PE, phosphatidylethanolamine; LPE, lysophosphatidylethanolamine; MMPE, monomethylphosphatidylethanolamine; PDPT, phosphatidyldimethylpropanethiol.

Lipid Species (C:N)	Observed *m*/*z*	Theoretical *m*/*z*	Error (ppm)	Fatty Acyl Chain (s)	Formula
PC(30:0)	706.5394	706.5387	0.9663	12:0-18:0	C_38_H_77_NO_8_P
**PC(30:3)**	700.4909	700.4917	−1.2501	*	C_38_H_71_NO_8_P
**PC(36:2)**	786.6019	786.6013	0.7575	18:1-18:1	C_44_H_85_NO_8_P
PC(36:3)	784.5872	784.5856	2.0557	*	C_44_H_83_NO_8_P
**PC(36:6)**	778.5397	778.5387	1.3224	22:6-14:0	C_44_H_77_NO_8_P
PC(37:2)	800.6168	800.6169	−0.1249	**	C_45_H_87_NO_8_P
PC(38:2)	814.6326	814.6326	−0.0365	18:1-20:1	C_46_H_89_NO_8_P
PC(38:5)	808.5837	808.5856	−4.3696	*	C_46_H_83_NO_8_P
PC(38:6)	806.5718	806.5700	2.2120	22:6-16:0; 18:2-20:4; 18:1-20:5	C_46_H_81_NO_8_P
PC(40:7)	832.5865	832.5856	1.0146	22:6-18:1	C_48_H_83_NO_8_P
PC(44:12)	878.5718	878.5700	2.1064	22:6-22:6	C_52_H_81_NO_8_P
LPC(14:0)	468.3092	468.3090	0.4532	**	C_22_H_47_NO_7_P
LPC(16:0)	496.3408	496.3403	0.9748	**	C_24_H_51_NO_7_P
LPC(18:1)	522.3567	522.3560	1.3845	**	C_26_H_53_NO_7_P
**LPC(22:6)**	568.3406	568.3403	0.4399	**	C_30_H_51_NO_7_P
PE(30:0)	664.4923	664.4917	0.9125	**	C_35_H_71_NO_8_P
PE(30:1)	662.4772	662.4761	1.7143	**	C_35_H_69_NO_8_P
**PE(30:3)**	658.4435	658.4448	−1.8979	*	C_35_H_65_NO_8_P
PE(31:1)†	676.4931	676.4917	2.0695	**	C_36_H_71_NO_8_P
PE(32:1)	690.5065	690.5074	−1.2180	16:0-16:1	C_37_H_73_NO_8_P
PE(32:2)	688.4952	688.4917	4.9833	**	C_37_H_71_NO_8_P
PE(32:6)	680.4258	680.4291	−4.8497	*	C_37_H6_3_NO_8_P
PE(34:1)	718.5382	718.5387	−0.7092	16:0-18:1; 14:0-20:1	C_39_H_77_NO_8_P
PE(34:2)	716.5239	716.5230	1.1764	*	C_39_H_75_NO_8_P
PE(34:3)	714.5087	714.5074	1.8055	*	C_39_H_73_NO_8_P
PE(34:4)	712.4914	712.4917	−0.4003	**	C_39_H_71_NO_8_P
**PE(36:2)**	744.5553	744.5543	1.2849	18:1-18:1	C_41_H_79_NO_8_P
PE(36:3)	742.5406	742.5387	2.5718	**	C_41_H_77_NO_8_P
PE(36:4)	740.5240	740.5230	1.2744	*	C_41_H_75_NO_8_P
PE(38:2)	772.5848	772.5856	−1.0554	*	C_43_H_83_NO_8_P
PE(38:5)	766.5373	766.5387	−1.8029	**	C_43_H_77_NO_8_P
PE(38:6)	764.5217	764.5230	−1.6806	*	C_43_H_75_NO_8_P
LPE(18:1)	480.3096	480.3090	1.2613	*	C_23_H_47_NO_7_P
MMPE (30:1)†	676.4931	676.4917	2.0695	**	C_36_H_71_NO_8_P
**PDPT (36:6)**	795.5009	795.4999	1.2571	**	C_44_H_76_O_8_PS
**PDPT (38:6)**	823.5327	823.5311	1.9429	**	C_46_H_80_O_8_PS
PDPT (40:7)	849.5469	849.5468	0.1177	**	C_48_H_82_O_8_PS
PDPT (44:12)	895.5312	895.5312	0.0558	*	C_52_H_80_O_8_PS

**Table 6:** Phospholipid species identified by liquid chromatography–mass spectrometry (LC–MS) in negative mode as [M – H]^–^ ions (error < 5 ppm). Polar head groups and fatty acyl composition were confirmed by tandem MS (MS/MS) spectra of [M-H]^−^ ions. The numbers in parentheses (C:N) indicate the number of carbon atoms (C) and double bonds (N) in fatty acyl chains. The most abundant species (relative abundance > 10%) are indicated in bold. Species identified only by retention time and mass accuracy are marked with an asterisk (*). PG, phosphatidylglycerol; PI, phosphatidylinositol.

Lipid Species (C:N)	Observed *m*/*z*	Theoretical *m*/*z*	Error (ppm)	Fatty Acyl Chains	Formula
PG(30:0)	693.4735	693.4707	4.0478	14:0-16:0	C_36_H_70_O_10_P
PG(30:1)	691.4569	691.4550	2.6592	14:0-16:1; 15:0-15:1; 16:0-14:1	C_36_H_68_O_10_P
PG(32:0)	721.5013	721.5020	−0.8607	17:0-15:0; 16:0-16:0; 18:0-14:0	C_38_H_74_O_10_P
PG(32:1)	719.4885	719.4863	3.0933	16:1-16:0, 14:0-18:1,15:0-17:1, 17:0-15:1	C_38_H_72_O_10_P
PG(32:2)	717.4738	717.4707	4.3987	16:1-16:1	C_38_H_70_O_10_P
**PG(34:1)**	747.5200	747.5176	3.2058	16:0-18:1, 16:1-18:0	C_40_H_76_O_10_P
**PG(34:2)**	745.5042	745.5020	2.9900	16:1-18:1	C_40_H_74_O_10_P
**PG(36:2)**	773.5355	773.5333	2.8853	18:1-18:1	C_42_H_78_O_10_P
PG(36:3)	771.5194	771.5176	2.2813	18:1-18:2	C_42_H_76_O_10_P
PG(36:7)	763.4559	763.4550	1.1658	20:5-16:2	C_42_H_68_O_10_P
PG(38:2)	801.5670	801.5646	3.0267	18:1-20:1, 19:1-19:1	C_44_H_82_O_10_P
**PI(32:7)**	795.4113	795.4085	3.5930	*	C_41_H_64_O_13_P
**PI(38:6)**	881.5196	881.5180	1.8331	16:0-22:6	C_47_H_78_O_13_P

**Table 7:** Sphingolipid species identified by liquid chromatography–mass spectrometry (LC–MS) in positive mode as [M + H]^+^ ions (error < 5 ppm). Identification as sphingolipids was confirmed by tandem MS (LC–MS/MS) spectra of [M+H]^+^ ions. C represents the total number of carbon atoms and N the total number of double bonds on fatty acyl chains. The most abundant species (relative abundance > 10%) are indicated in bold. Species identified only by retention time and mass accuracy are marked with an asterisk (*). sGSL, sialic acid glycosphingolipid; hGSL, host glycosphingolipid; Cer, ceramide.

Lipid Species (C:N)	Observed *m*/*z*	Theoretical *m*/*z*	Error (ppm)	Fatty Acyl Chain (s)	Formula
**sGSL (d40:2)**	870.6685	870.6670	1.7228	d18:2/22:0	C_49_H_92_O_11_N
**sGSL (d40:1)**	872.6829	872.6827	0.2292	d18:1/22:0	C_49_H_94_O_11_N
**hGSL**	806.6159	806.6146	1.6117	d19:3/h22:2	C_47_H_84_O_9_N
Cer(d36:2)	564.5363	564.5356	1.3198	*	C_36_H_70_NO_3_
**Cer(d38:1)**	594.5827	594.5825	0.2838	*	C_38_H_76_NO_3_
**Cer(d38:2)**	592.5672	592.5669	0.6268	*	C_38_H_74_NO_3_
**Cer(d40:1)**	622.6143	622.6138	0.7211	d18:1/22:0	C_40_H_80_NO_4_
**Cer(d40:2)**	620.5975	620.5982	−1.0393	d18:2/22:0	C_40_H_78_NO_5_

## Supplementary Materials

The following are available online at https://www.mdpi.com/2218-273X/10/10/1434/s1, Supplementary Table S1. In-house database used for peak identification in MZmine (positive mode), Supplementary Table S2. In-house database used for peak identification in MZmine (negative mode), Figure S1. Representative examples of LC–MS chromatograms obtained by analysis of the polar lipids extracts from *Emiliania huxleyi*, acquired on (a) positive mode and (b) negative mode, Figure S2: Relative abundance of acidic and neutral glycolipids identified by LC–MS from *Emiliania huxleyi*, Figure S3: LC–MS/MS spectrum of sulfoquinovosyldiacylglycerol SQDG (30:0) species observed in negative mode as [M − H]^−^ ion at *m*/*z* 765.48, Figure S4: LC–MS/MS spectrum of digalactosyldiacylglycerol DGDG (32:1) species observed in positive mode as [M + NH_4_]^+^ ion at *m*/*z* 908.63, Figure S5: Relative abundance of betaine lipid species identified by LC–MS from *Emiliania huxleyi,* Figure S6: LC–MS/MS spectrum of diacylglyceryl-N,N,N-trimethyl-homoserine DGTS (36:2) species observed in positive mode as [M + H]^+^ ion at *m*/*z* 764.68, Figure S7: LC–MS/MS spectra of diacylglyceryl carboxyhydroxymethylcholine DGCC(36:6) species observed (a) in positive mode as [M + H]^+^ ion at *m*/*z* 772.57 and (b) in negative mode as [M + CH_3_COO]^−^ ion at *m*/*z* 830.58, Figure S8: LC–MS/MS spectrum of betaine like lipid BLL (38:6) species observed in positive mode as [M + H]^+^ ion at *m*/*z* 830.58, Figure S9: Relative abundance of phospholipids species identified by LC–MS from *Emiliania huxleyi,* Figure S10: LC–MS/MS spectra of phosphatidylcholine PC (36:6) species observed (a) in positive mode as [M+H]^+^ ion at *m*/*z* 778.54 and (b) in negative mode as [M + CH_3_COO]^−^ ion at *m*/*z* 836.54, Figure S11: LC–MS/MS spectra of phosphatidylethanolamine PE (34:1) species observed in (a) positive mode as [M + H]^+^ ion at *m*/*z* 718.47 and (b) in negative mode as [M − H]^−^ ion at *m*/*z* 716.48, Figure S12: (a) Extracted ion current chromatogram of isomeric species, phosphatidylethanolamine PE (31:1) and monomethylphosphatidylethanolamine MMPE (30:1) species, observed in positive mode as [M + H]^+^ ions at *m*/*z* 676.49. LC–MS/MS spectra of respective [M + H]^+^ ion of (B) PE (31:1) (retention time: 4.33 min) and (C) MMPE (30:1) (retention time: 5.22 min), Figure S13: LC–MS/MS spectrum of phosphatidyldimethylpropanethiol PDPT (36:6) species observed in positive mode as [M + H]^+^ ion at *m*/*z* 795.50, Figure S14: LC–MS/MS spectrum of phosphatidylglycerol PG (32:1) species observed in negative mode as [M − H]^−^ ion at *m*/*z* 719.49, Figure S15: LC–MS/MS spectrum of phosphatidylinositol PI (38:6) species observed in negative mode as [M − H]^−^ ion at *m*/*z* 881.52, Figure S16: Relative abundance of sphingolipid species identified by LC–MS from *Emiliania huxleyi*: (a) ceramides (Cer) and (b) sialic acid glycosphingolipids (sGSL), Figure S17: LC–MS/MS spectra of sialic acid glycosphingolipids sGSL (d18:1/22:0) species observed (a) in positive mode as [M + H]^+^ ion at *m*/*z* 872.68 and (b) in negative mode as [M − H]^−^ ion at *m*/*z* 870.67, Figure S18: LC–MS/MS spectrum of host glycosphingolipid hGSL (d19:3/h22:2) species observed in positive mode as [M + H]^+^ ion at *m*/*z* 806.62, Figure S19: LC–MS/MS spectrum of ceramide Cer (d18:2/22:0) species observed in positive mode as [M + H]^+^ ion at *m*/*z* 620.40.
