# Supplementary material for: The Polar Lipidome of Cultured Emiliania huxleyi: A Source of Bioactive Lipids with Relevance for Biotechnological Applications
Source: Biomolecules. 2020 Oct 12;10(10):1434. doi: 10.3390/biom10101434 (PMC7650762; doi:10.3390/biom10101434)
Supplement: Supplementary file 1 [file biomolecules-10-01434-s001.zip › Emiliania_huxleyi polar lipidome_supplementary_material.pdf]

biomolecules

MDPI

Article

The Polar Lipidome of Cultured *Emiliania huxleyi*: A source of Bioactive Lipids with Relevance for Biotechnological Applications

Susana S. Aveiro [1], Tânia Melo [2], Ana Figueiredo [1], Pedro Domingues [1], Hugo Pereira [3], Inês B. Maia [4], Joana Silva [5], M. Rosário Domingues [1,2], Cláudia Nunes [6] and Ana S. P. Moreira [1,6,*]

[1] Mass Spectrometry Center, LAQV-REQUIMTE, Department of Chemistry, University of Aveiro, Campus Universitário de Santiago, 3810-193 Aveiro, Portugal; s.aveiro@ua.pt (S.S.A.), ana90@ua.pt (A.F.), p.domingues@ua.pt (P.D.), mrd@ua.pt (M.R.D.); ana.moreira@ua.pt (A.M.)

[2] ECOMARE, CESAM - Centre for Environmental and Marine Studies, Department of Chemistry, University of Aveiro, Campus Universitário de Santiago, 3810-193 Aveiro, Portugal; taniamelo@ua.pt

[3] Green Colab - Associação Oceano Verde, University of Algarve, Campus de Gambelas, 8005-139, Portugal; galvaohugo@gmail.com

[4] Centre of Marine Sciences, University of Algarve, Campus de Gambelas, 8005-139 Faro, Portugal; ibmaia@ualg.pt

[5] Allmicroalgae Natural Products S.A., Apartado 9, 2449-909 Pataias, Portugal; joana.g.silva@allmicroalgae.com

[6] CICECO - Aveiro Institute of Materials, Department of Chemistry, University of Aveiro, Campus Universitário de Santiago, 3810-193 Aveiro, Portugal; claudianunes@ua.pt

* Correspondence: ana.moreira@ua.pt; Tel.: +351-234-401-505

Supplementary Materials

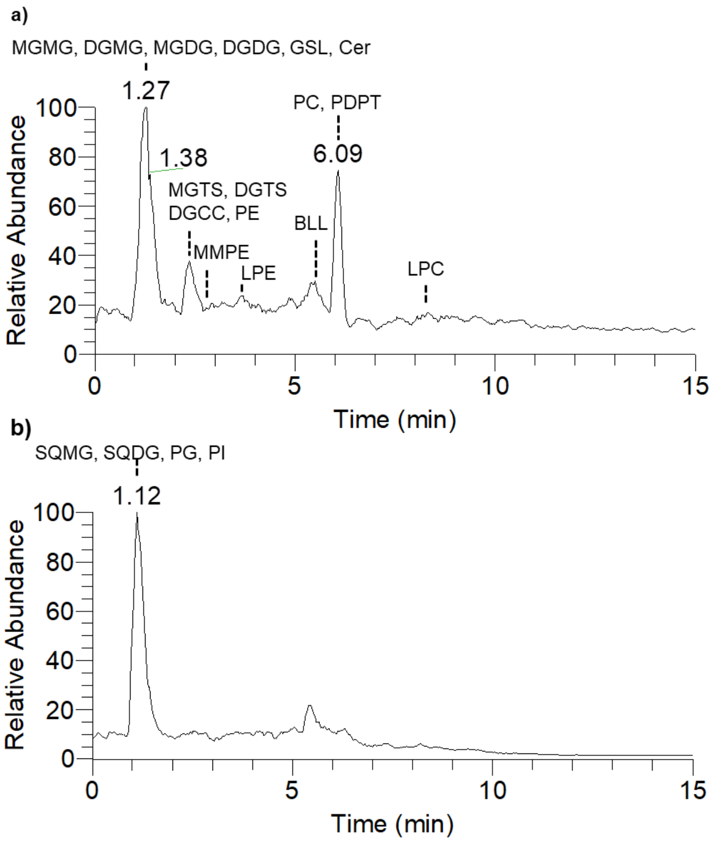

Supplementary Figure S1. Representative examples of LC-MS chromatograms obtained by analysis of the polar lipids extracts from *Emiliania huxleyi*, acquired on (a) positive mode and (b) negative mode.

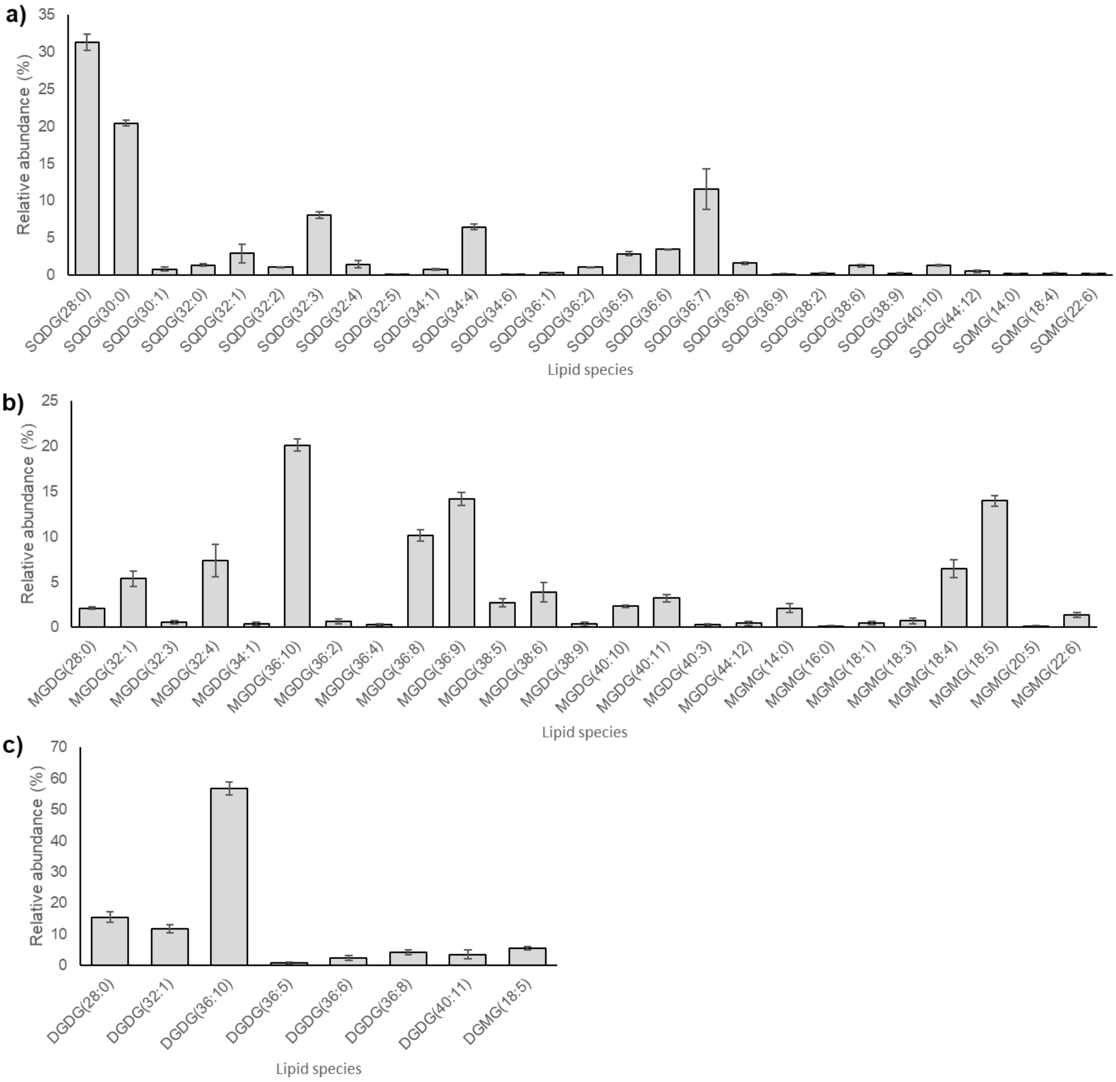

Supplementary Figure S2. Relative abundance of a) acidic and (b and c) neutral glycolipids identified by LC-MS from *Emiliania huxleyi*: (a) sulfoquinovosyldiacylglycerol (SQDG) and its lyso form, sulfoquinovosylmonoacylglycerol (SQMG); (b) monogalactosyldiacylglycerol (MGDG) and its lyso form, monogalactosylmonoacylglycerol (MGMG); (c) digalactosyldiacylglycerol (DGDG) and its lyso form, digalactosylmonoacylglycerol (DGMG).

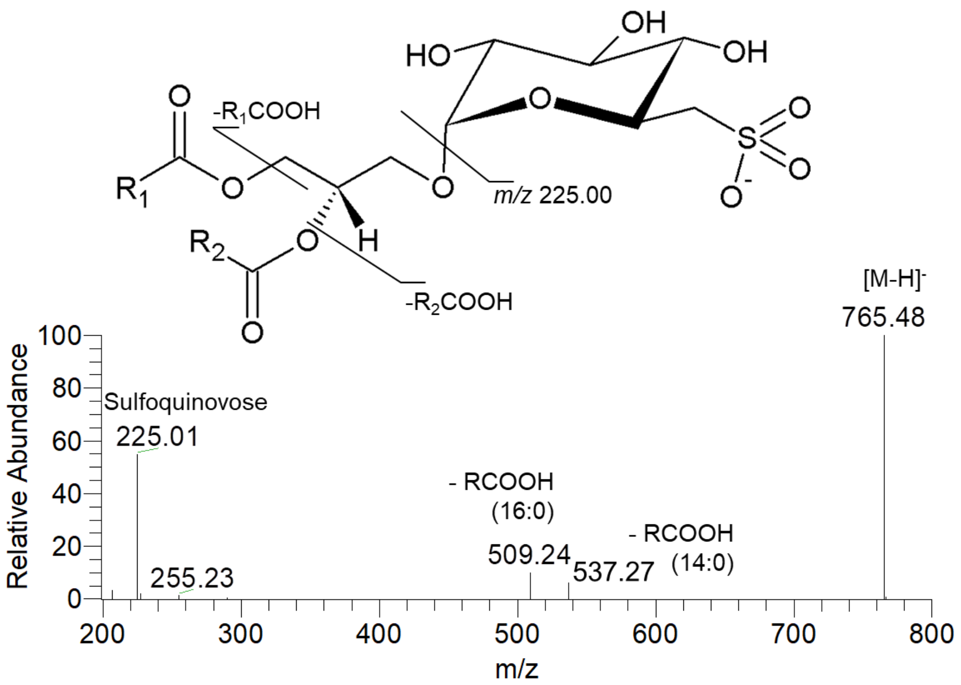

Supplementary Figure S3. LC-MS/MS spectrum of sulfoquinovosyldiacylglycerol SQDG (30:0) species observed in negative mode as [M-H]- ion at *m*/*z* 765.48. The main fragmentation pathways and product ions are shown in the structure of the SQDG: the anion of the sulfoquinovosyl polar head group ($C_6H_9O_4SO_3^-$) at *m*/*z* 225.01 confirms the identification as sulfolipid; the product ions at *m*/*z* 509.24 and 537.27, resulting from neutral loss of palmitic acid (16:0) (−256 Da) and myristic acid (14:0) (−228 Da), respectively, allow the identification in terms of fatty acyl composition as SQDG (14:0-16:0).

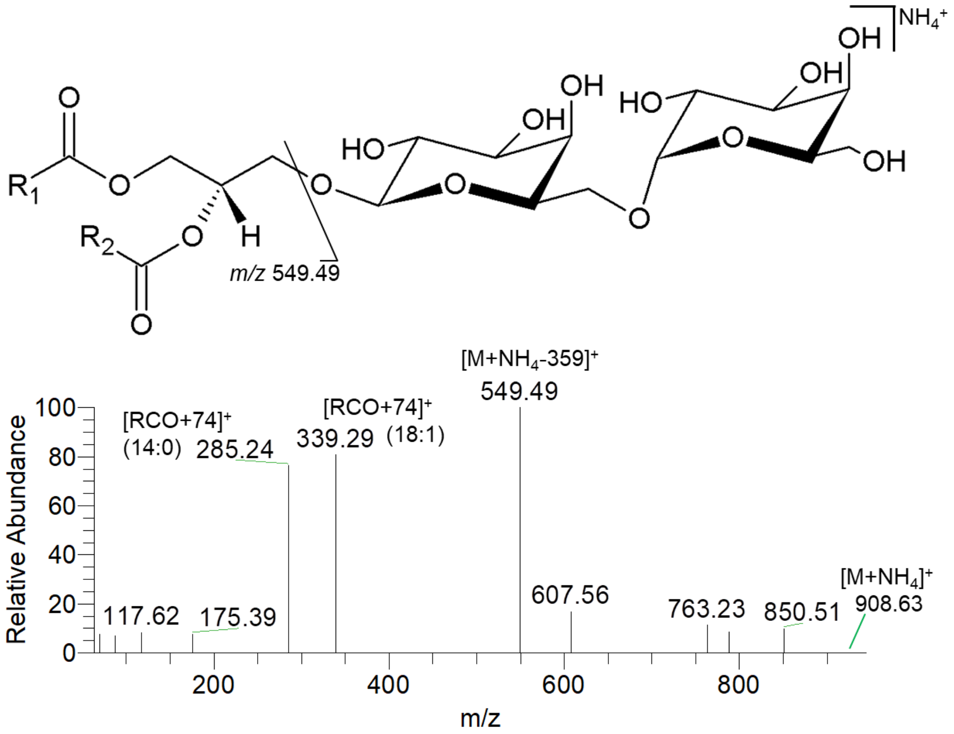

Supplementary Figure S4. LC-MS/MS spectrum of digalactosyldiacylglycerol DGDG (32:1) species observed in positive mode as $[M+NH_4]^+$ ion at *m/z* 908.63. The main fragmentation pathways and product ions are shown in the structure of the DGDG: the product ion observed at *m/z* 549.49 (- 359 Da), resulting from a combined loss of NH_3 (-17 Da) and carbohydrate moiety (-180 Da plus 162 Da), corroborates the presence of a digalactosyl unit in the polar head; the product ions at *m/z* 285.24 and 339.29, corresponding to acylium ions plus 74 Da of the glycerol backbone (RCO + 74 Da) of fatty acids 14:0 and 18:1, respectively, support the identification in terms of fatty acyl composition as DGDG (14:0-18:1).

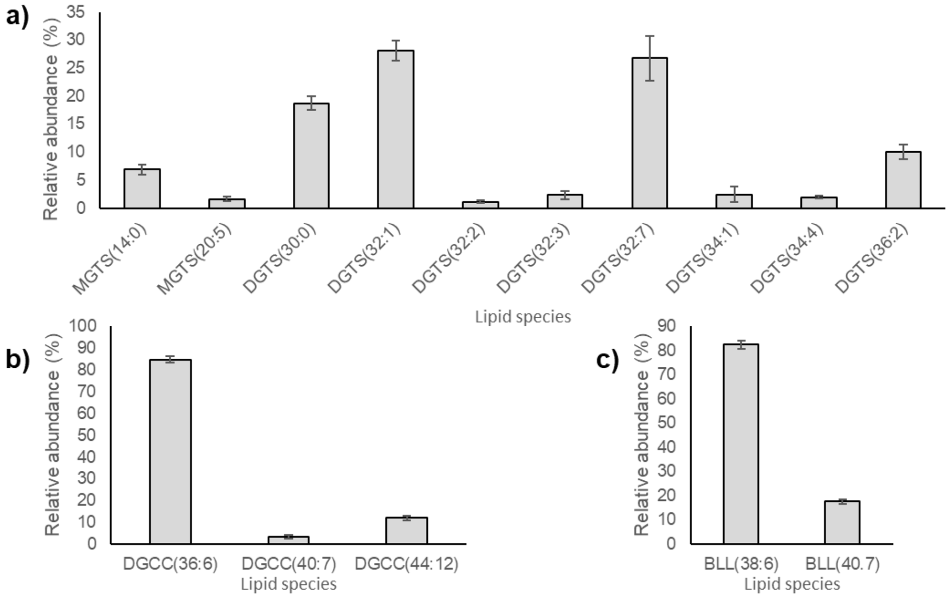

Supplementary Figure S5. Relative abundance of betaine lipid species identified by LC-MS from *Emiliania huxleyi*: a) monoacyl and diacylglyceryl-N,N,N-trimethyl homoserine (MGTS and DGTS); b) diacylglyceryl carboxyhydroxymethylcholine (DGCC); and c) betaine like lipids (BLL).

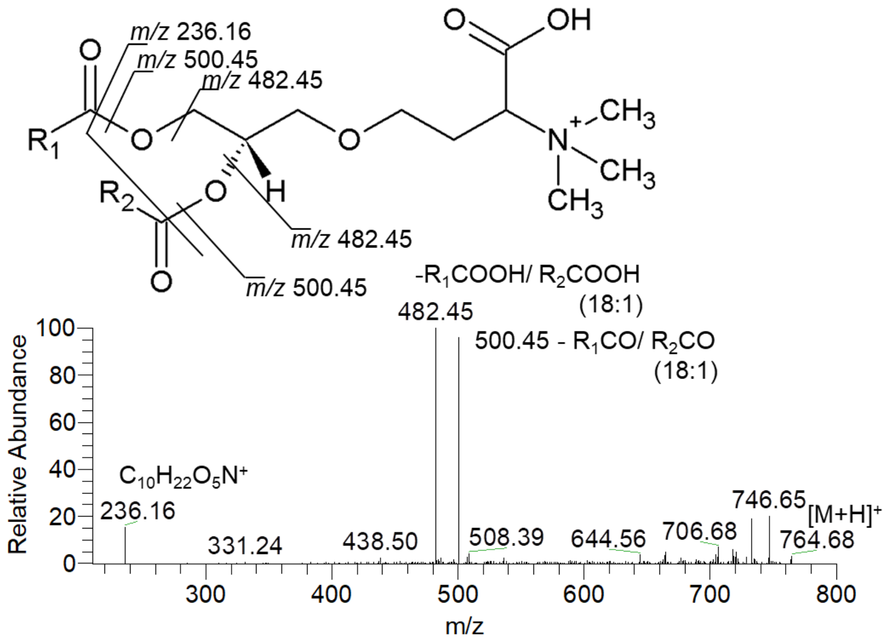

Supplementary Figure S6. LC-MS/MS spectrum of diacylglyceryl-N,N,N-trimethyl-homoserine DGTS (36:2) species observed in positive mode as $[M+H]^+$ ion at *m/z* 764.68. The main fragmentation pathways and product ions are shown in the structure of the DGTS: the product ion at *m/z* 236.16 ($C_{10}H_{22}O_5N^+$), resulting from the combined loss of the two fatty acids as ketene derivatives (R_1CO+R_2CO), corroborates N,N,N-trimethyl-homoserine as polar head; the product ions observed at *m/z* 500.45 and 482.45, corresponding to the loss of 18:1 as ketene (-264 Da) and acid (-282 Da) derivatives, allow the identification in terms of the fatty acyl composition as DGTS (18:1-18:1).

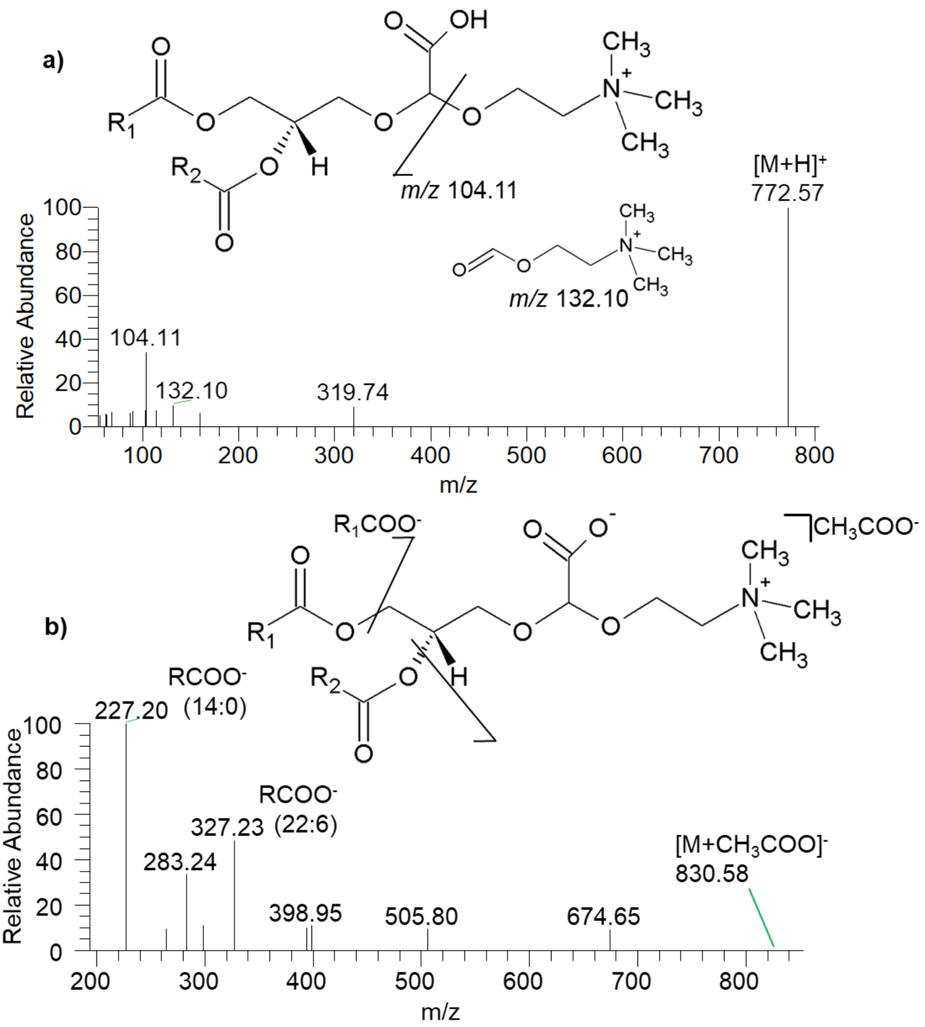

Supplementary Figure S7. LC-MS/MS spectra of diacylglyceryl carboxyhydroxymethylcholine DGCC(36:6) species observed a) in positive mode as $[M+H]^+$ ion at *m/z* 772.57 and b) in negative mode as $[M+CH_3COO]^-$ ion at *m/z* 830.58. The main fragmentation pathways and product ions are shown in the structure of the DGTS: the product ions at *m/z* 104.11 ($C_5H_{14}NO^+$) and *m/z* 132.10 ($C_6H_{14}NO_2^+$), in positive mode (a), corroborate the identification of the DGCC polar head group; in negative mode (b), the product ions at *m/z* 227.20 and *m/z* 327.23 correspond to the fatty acyl carboxylate anions of 14:0 and 22:6, which allow the identification in terms of the fatty acyl composition as DGCC (14:0-22:6).

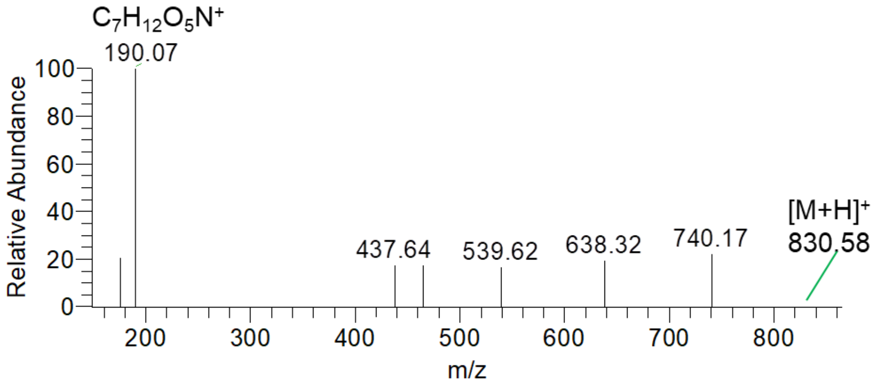

Supplementary Figure S8. LC-MS/MS spectrum of betaine like lipid BLL (38:6) species observed in positive mode as $[M+H]^+$ ion at *m/z* 830.58. The product ion observed at *m/z* 190.07 ($C_7H_{12}O_5N^+$) confirms the identification as BLL.

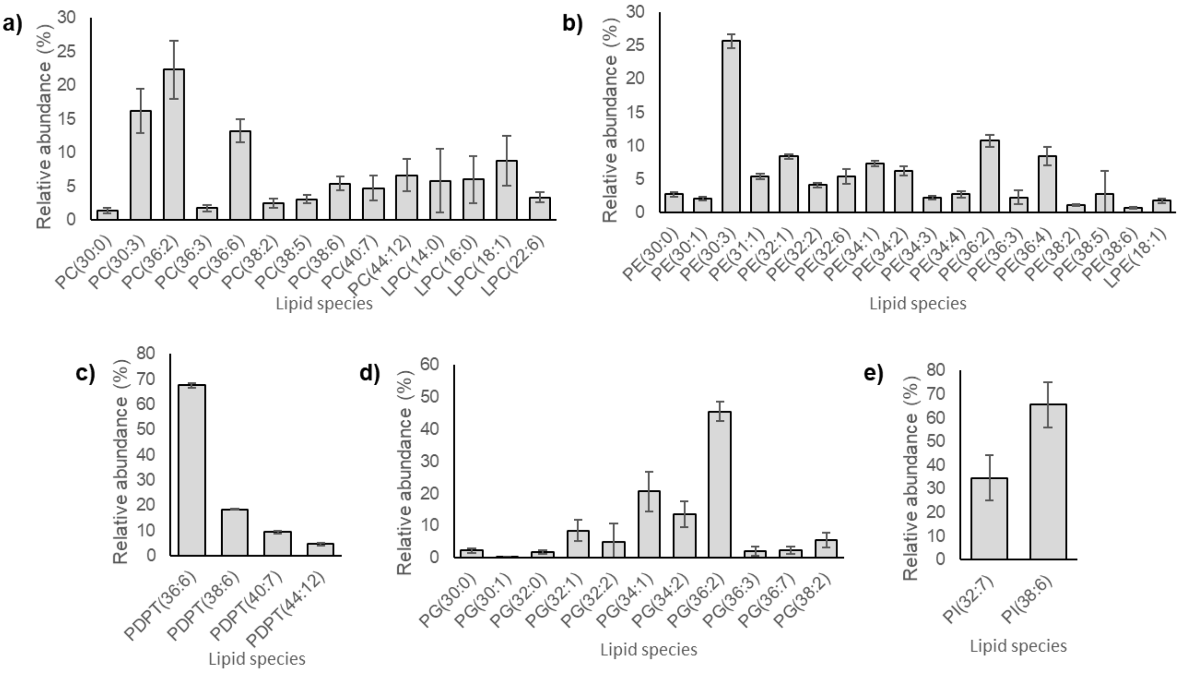

Supplementary Figure S9. Relative abundance of phospholipids species identified by LC-MS from *Emiliania huxleyi*: (a) phosphatidylcholine (PC) and its lyso form, lysophosphatidylcholine (LPC); (b) phosphatidylethanolamine (PE) and its lyso form, lysophosphatidylethanolamine (LPE); (c) phosphatidyldimethylpropanethiol (PDPT); (d) phosphatidylglycerol (PG) and (e) phosphatidylinositol (PI).

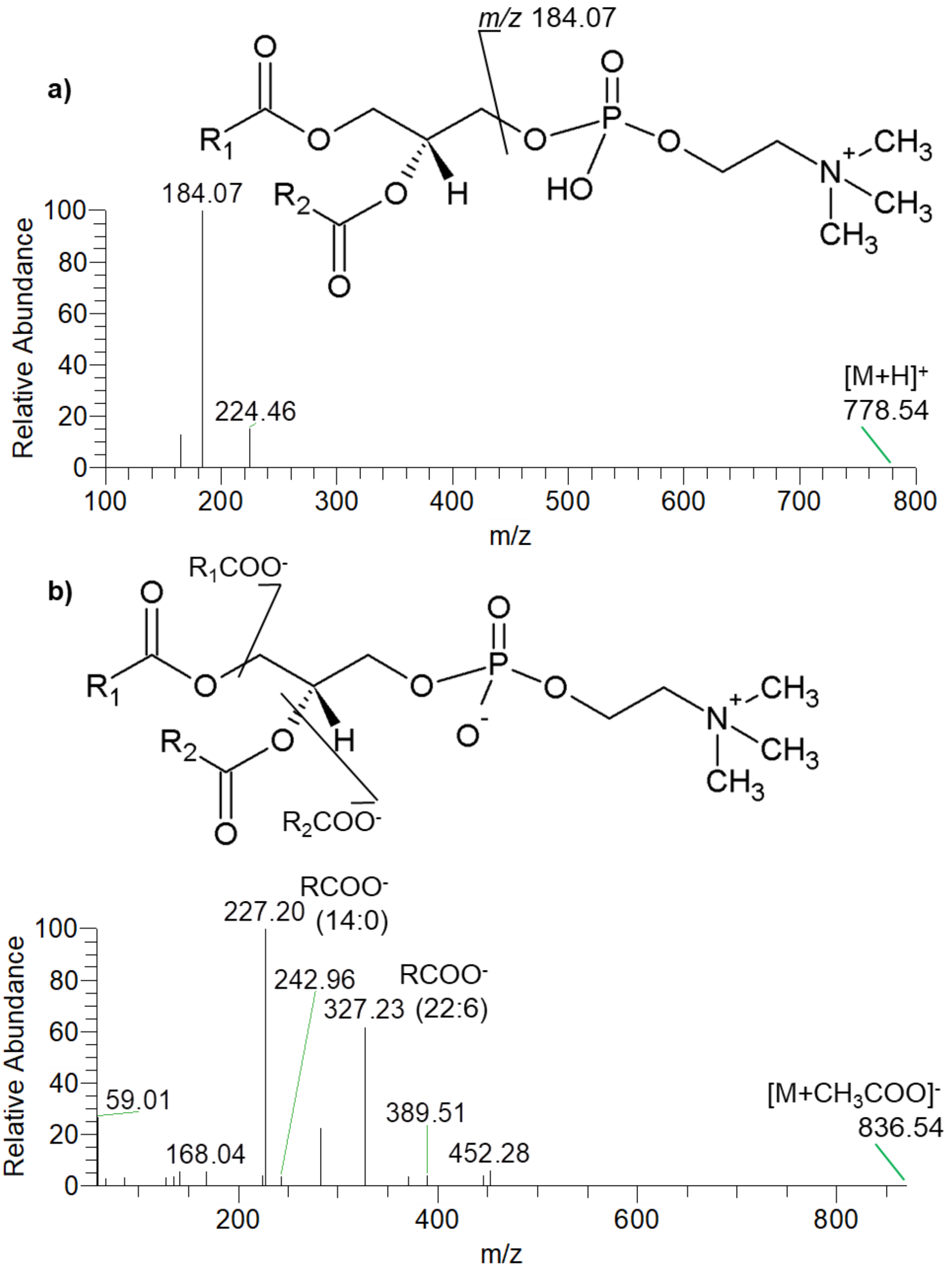

Supplementary Figure S10. LC-MS/MS spectra of phosphatidylcholine PC (36:6) species observed (a) in positive mode as $[M+H]^+$ ion at *m/z* 778.54 and (b) in negative mode as $[M+CH_3COO]^-$ ion at *m/z* 836.54. The main fragmentation pathways and product ions are shown in the structure of the PC: the product ion observed at *m/z* 184.07, in positive mode (a), corresponds to the phosphocholine anion ($C_5H_{15}NO_4P^+$) and corroborates the identification as PC; the product ions at *m/z* 227.20 and *m/z* 327.23, in negative mode (b), correspond to fatty acyl carboxylate anions of 14:0 and 22:6, respectively, and allow the identification of the fatty acyl composition of PC (14:0-22:6).

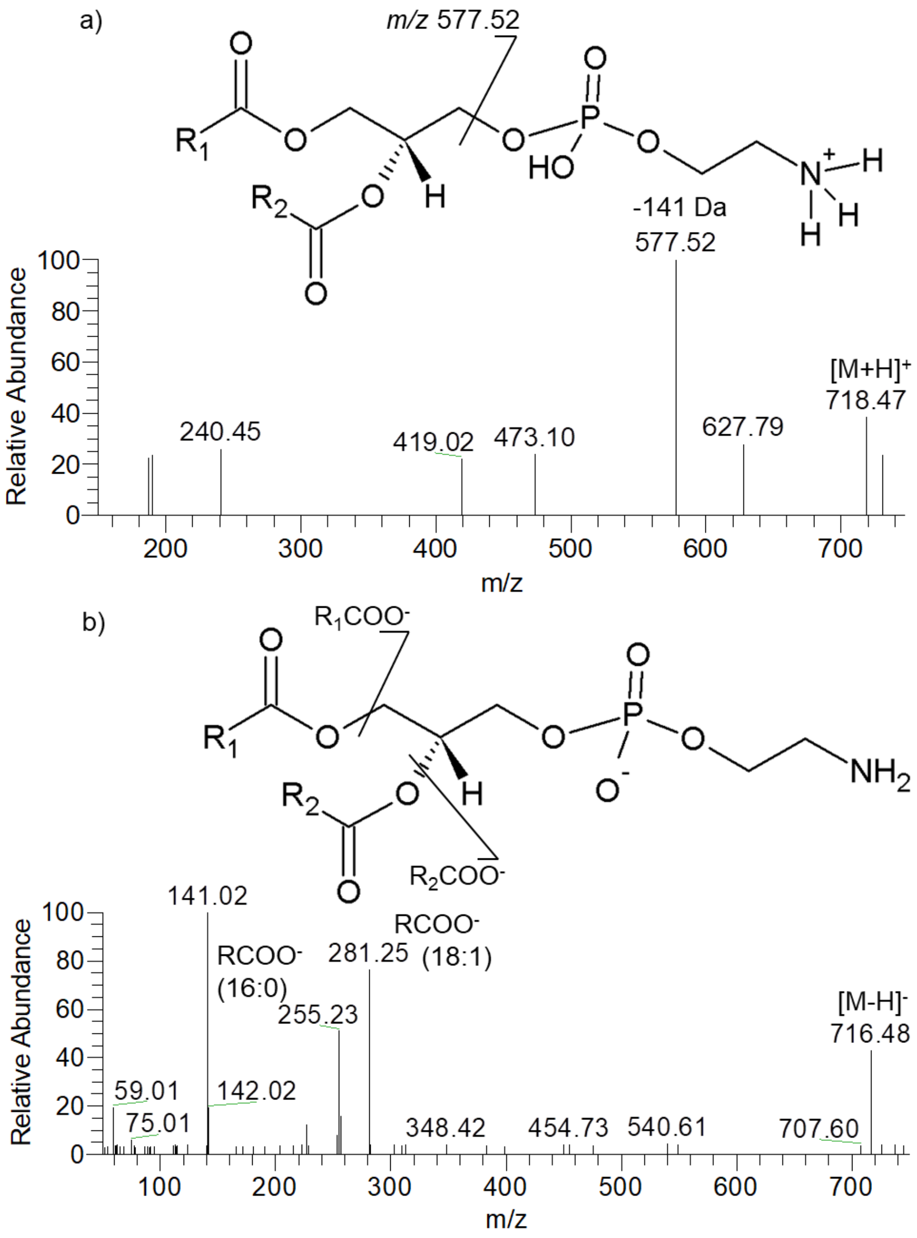

Supplementary Figure S11. LC-MS/MS spectra of phosphatidylethanolamine PE (34:1) species observed in (a) positive mode as $[M+H]^+$ ion at *m/z* 718.47 and (b) in negative mode as $[M-H]^-$ ion at *m/z* 716.48. The main fragmentation pathways and product ions are shown in the structure of the PE: the product ion observed at *m/z* 577.52, in positive mode (a), resulted from the characteristic neutral loss of polar head group (-141 Da) and corroborates the identification as PE; the product ions at *m/z* 255.23 and *m/z* 281.25, in negative mode (b), correspond to fatty acyl carboxylate anions of 16:0 and 18:1, respectively, and allow the identification of the fatty acyl composition of PE (16:0-18:1).

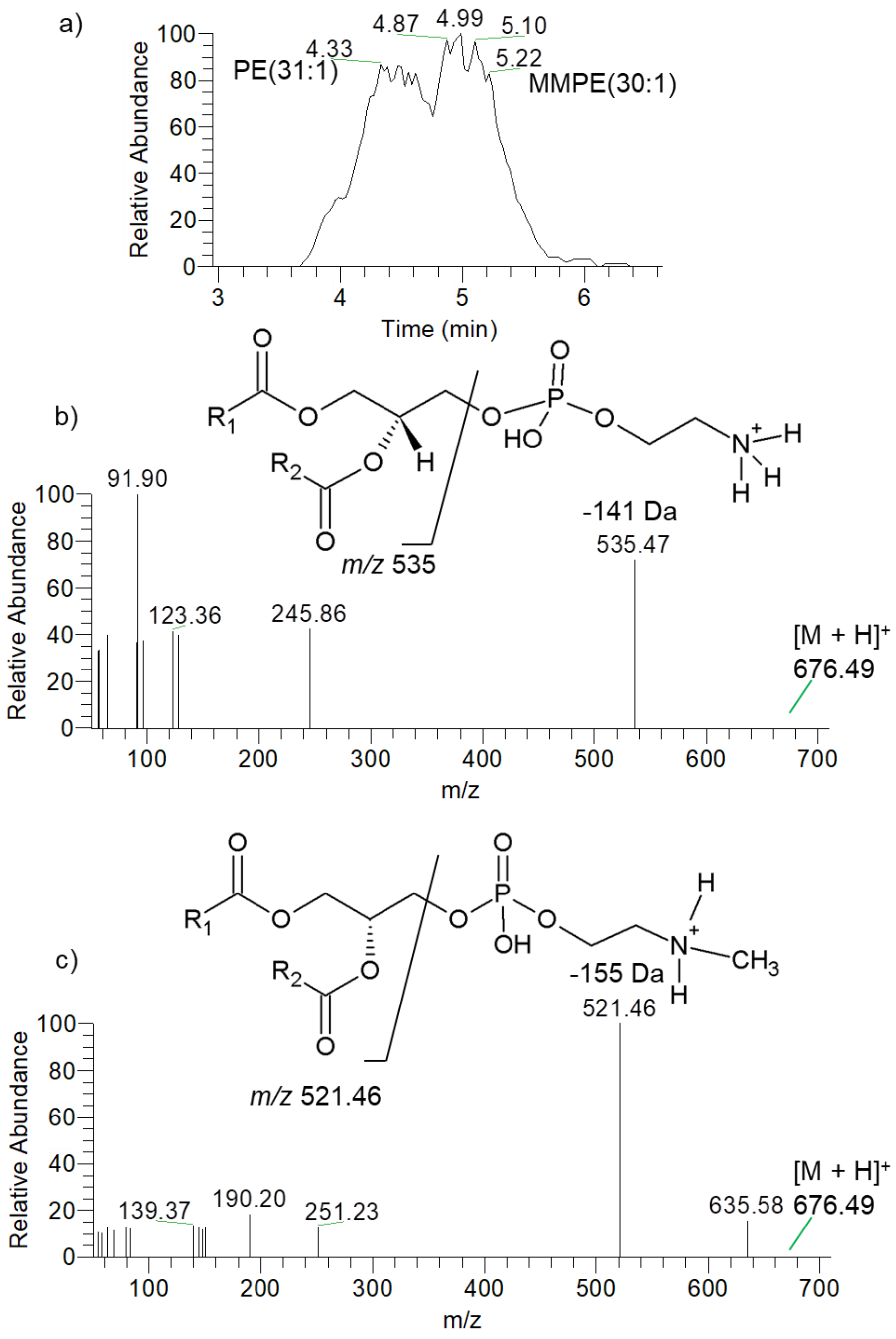

Supplementary Figure S12. (a) Extracted ion current chromatogram of isomeric species, phosphatidylethanolamine PE (31:1) and monomethylphosphatidylethanolamine MMPE (30:1) species, observed in positive mode as $[M+H]^+$ ions at *m*/*z* 676.49 . LC-MS/MS spectra of respective $[M+H]^+$ ion of (b) PE (31:1) (retention time: 4.33 min) and (c) MMPE (30:1) (retention time: 5.22 min).

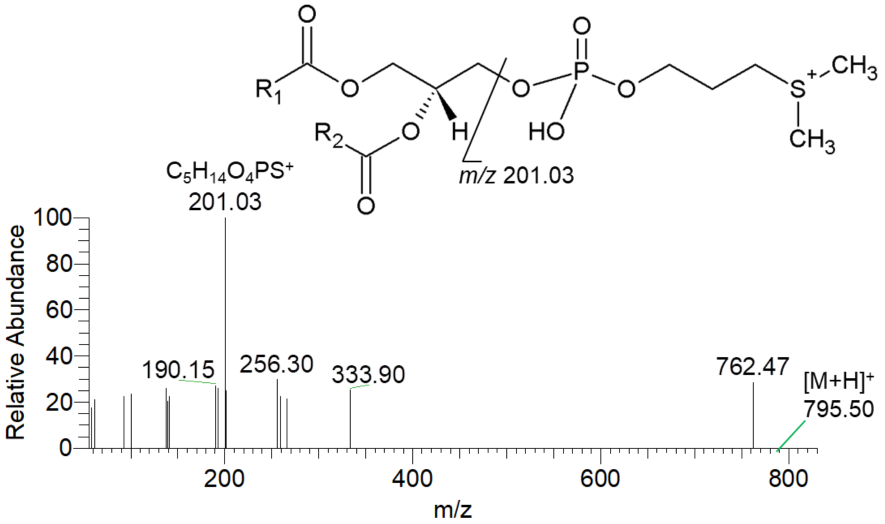

Supplementary Figure S13. LC-MS/MS spectrum of phosphatidyldimethylpropanethiol PDPT (36:6) species observed in positive mode as $[M+H]^+$ ion at *m/z* 795.50. The main fragmentation pathway of polar head group and product ion is shown in the structure of the PDPT: the product ion observed at *m/z* 201.03 ($C_5H_{14}O_4PS^+$), corresponding to the PDPT polar head, corroborates the identification as PDPT.

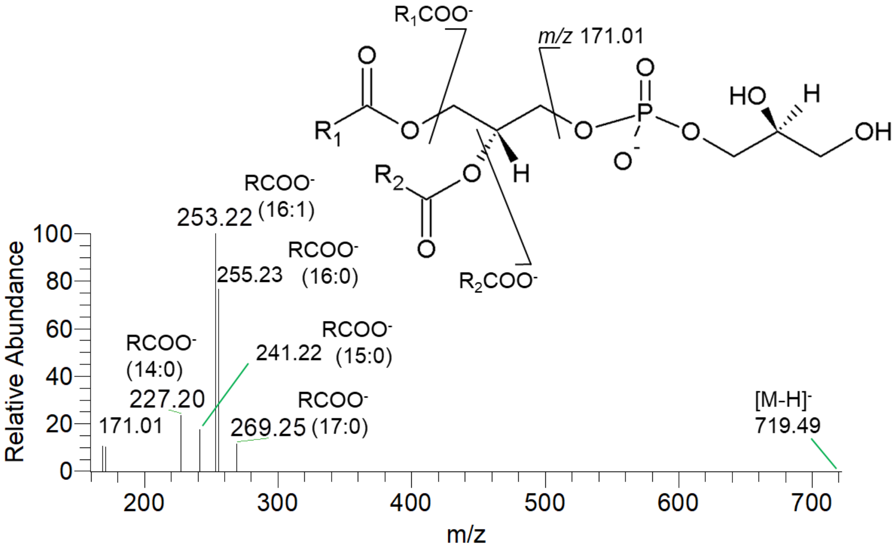

Supplementary Figure S14. LC-MS/MS spectrum of phosphatidylglycerol PG (32:1) species observed in negative mode as $[M-H]^-$ ion at *m/z* 719.49. The main fragmentation pathways and product ions are shown in the structure of the PG: the product ion at *m/z* 171.01 ($C_3H_7O_2OPO_3H$), corresponding to the glycerol phosphate anion, corroborates the identification as PG species; the product ions *m/z* 227.20, 253.22, 255.23, 241.22 and 269.25 correspond to fatty acyl carboxylate anions of 14:0, 15:0, 16:1, 16:0 and 17:0 respectively, and allow to identify different fatty acyl compositions of PG (32:1), namely 14:0-18:1,15:0-17:1, 17:0-15:1.

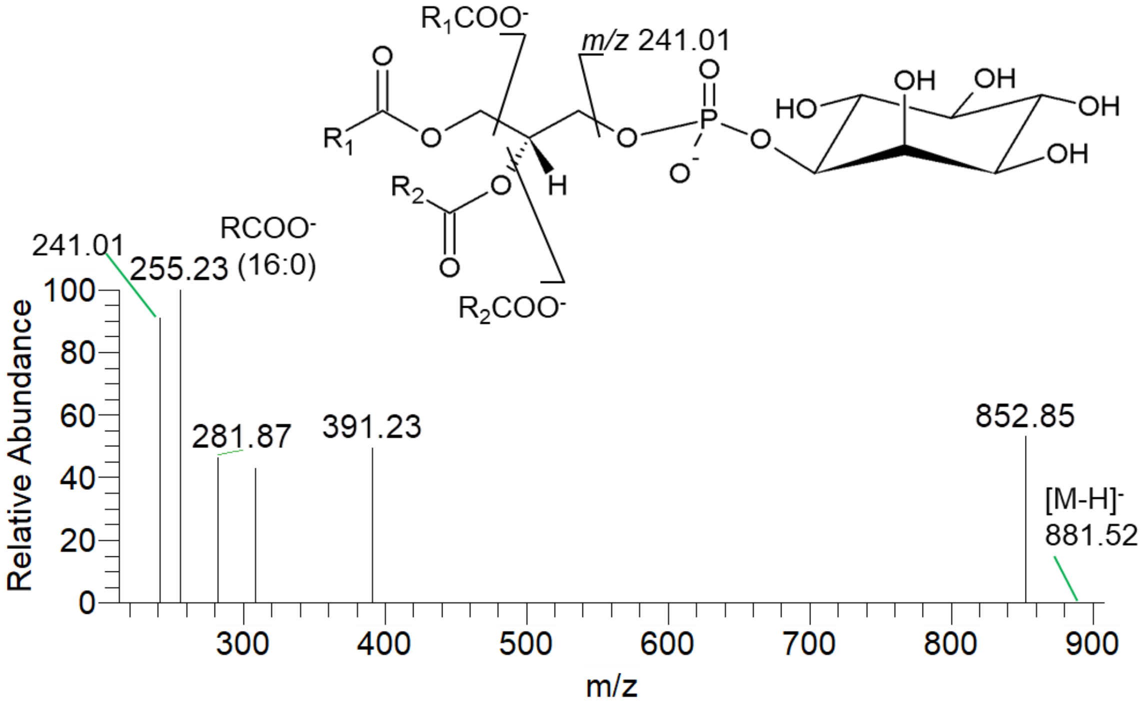

Supplementary Figure S15. LC-MS/MS spectrum of phosphatidylinositol PI (38:6) species observed in negative mode as [M-H]$^-$ ion at *m/z* 881.52. The main fragmentation pathways and product ions are shown in the structure of the PI: the product ion at *m/z* 241.01 ($C_6H_{10}O_5PO_3$), corresponding to an inositol-1,2-cyclic phosphate anion, corroborates the identification as PI species; the product ion observed at *m/z* 255.23, corresponding to fatty acyl carboxylate anion of 16:0, allows to infer the fatty acyl composition as PI (16:0-22:6).

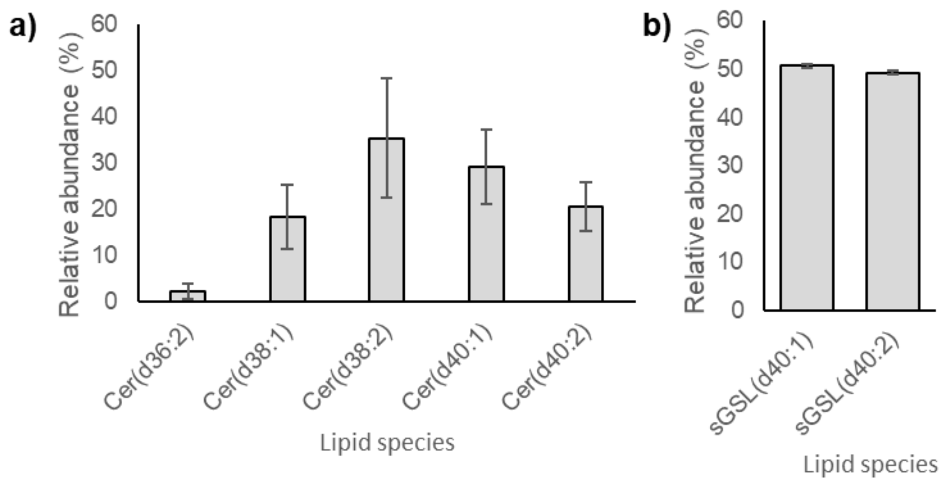

Supplementary Figure S16. Relative abundance of sphingolipid species identified by LC-MS from *Emiliania huxleyi*: (a) ceramides (Cer) and (b) sialic acid glycosphingolipids (sGSL).

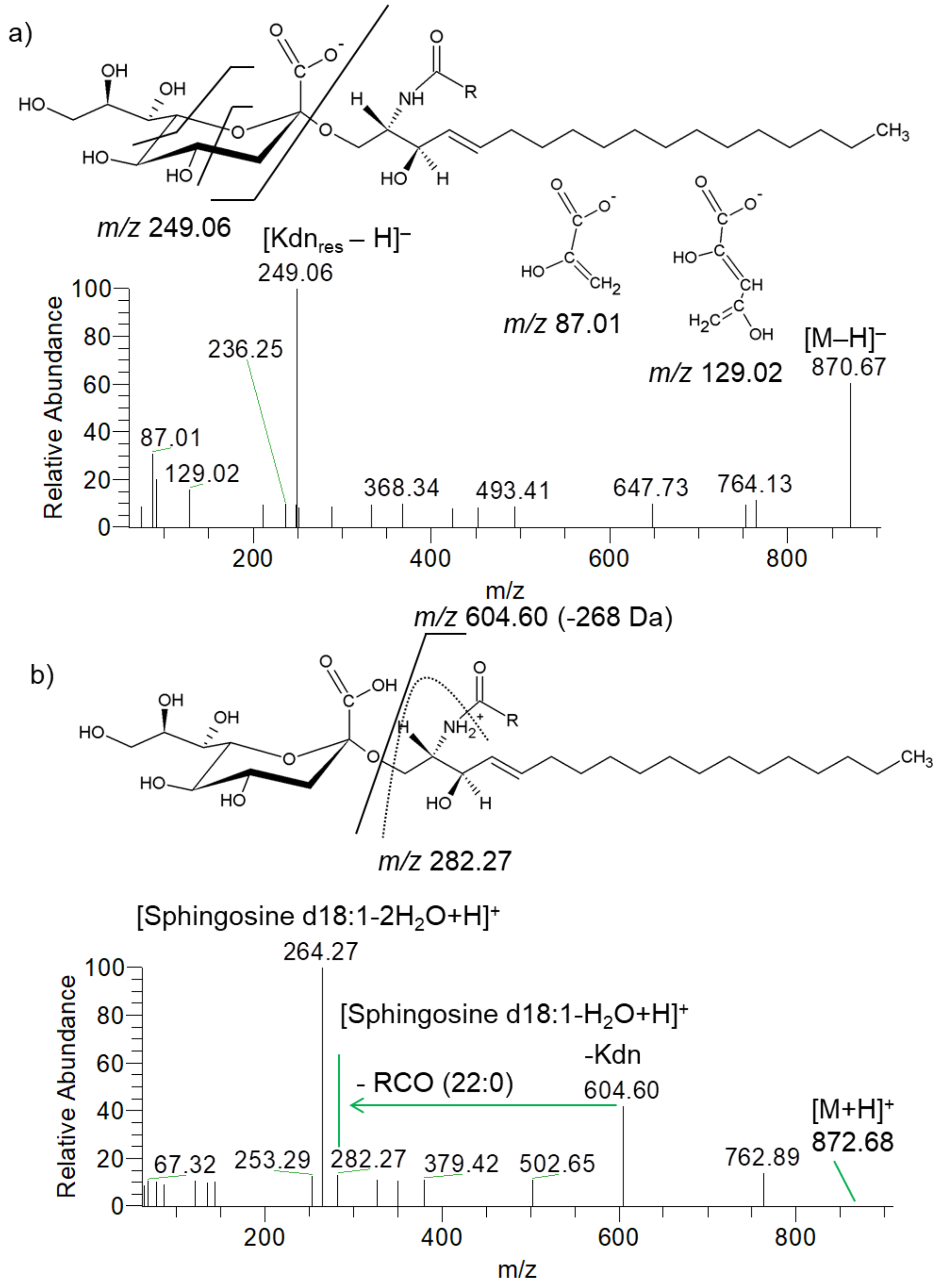

Supplementary Figure S17. LC-MS/MS spectra of sialic acid glycosphingolipids sGSL (d18:1/22:0) species observed a) in positive mode as [M+H]$^+$ ion at *m/z* 872.68 and b) in negative mode as [M-H]$^-$ ion at *m/z* 870.67. The main fragmentation pathways and product ions are shown in the structure of the sGSL: the product ion observed, in negative mode (a), at *m/z* 249.06 ($C_9H_{13}O_8$), attributed to a single sialic acid 2-keto-3-deoxynononic acid residue (Kdn$_{res}$), corroborates the presence of Kdn in the sGSL head group; the two product ions observed in negative mode at *m/z* 129.02 ($C_5H_5O_4$) and 87.01 ($C_3H_3O_3$), result from cross-ring cleavages of the Kdn; the product ions observed in positive mode (b) at *m/z* 264.27 and 282.27, corroborates the presence of sphingosine d18:1; the product ion observed in positive mode (b) at *m/z* 604.60 results from a neutral loss of Kdn and supports the identification as sGSL; the fatty acyl composition was found by the mass difference (322 Da) between product ions observed in positive mode (b) at *m/z* 604.60 and 282.27, corresponding to the fatty acid 22:0 as a ketene derivative.

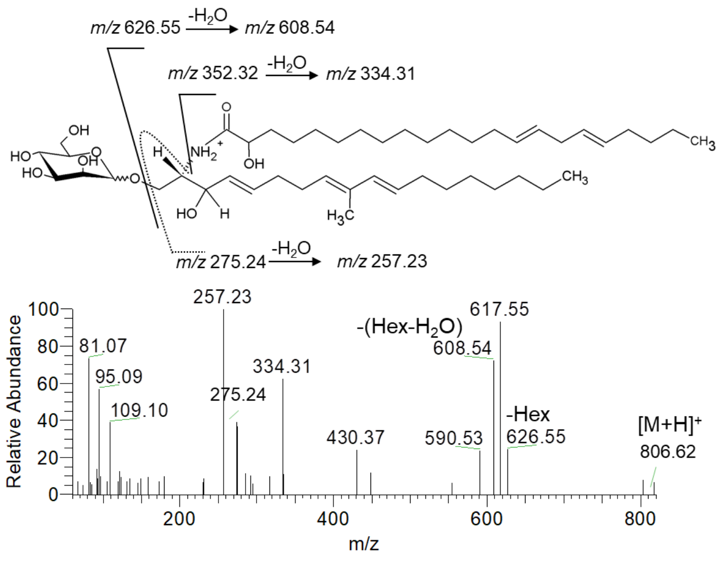

Supplementary Figure S18. LC-MS/MS spectrum of host glycosphingolipid hGSL (d19:3/h22:2) species observed in positive mode as $[M+H]^+$ ion at *m/z* 806.62. The main fragmentation pathways and product ions are shown in the structure of the hGSL: the product ions observed at *m/z* 626.55(-180 Da) and 608.54 (-198 Da), result from glycosidic cleavage with neutral loss of an hexose (Hex) and an additional loss of water, respectively; an amino fatty acid product ion (*m/z* 334.31) and long-chain base product ions (*m/z* 275.24 and 257.23) were also observed.

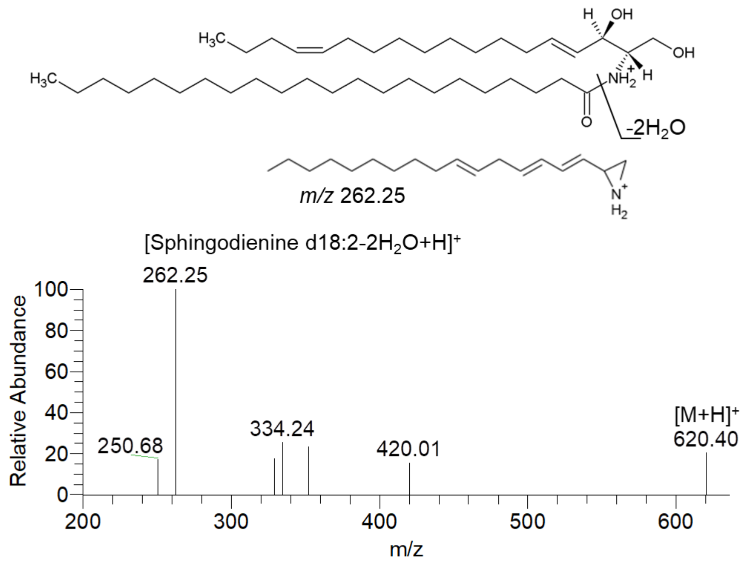

Supplementary Figure S19. LC-MS/MS spectrum of ceramide Cer (d18:2/22:0) species observed in positive mode as $[M+H]^+$ ion at *m/z* 620.40. The main fragmentation pathway and product ion are shown in the structure of the Cer: the product ion observed at *m/z* 262.25, corresponding to [Sphingodienine d18:2-$2H_2O$+H]$^+$, corroborates the identification as ceramide.
